# Self-control, Mental Health Problems, and Family Functioning in Adolescence and Young Adulthood: Between-person Differences and Within-person Effects

**DOI:** 10.1007/s10964-021-01564-3

**Published:** 2022-01-18

**Authors:** Yugyun Kim, Jennifer S. Richards, Albertine J. Oldehinkel

**Affiliations:** Interdisciplinary Center Psychopathology and Emotion regulation, Department of Psychiatry, University of Groningen, University Medical Center Groningen, Groningen, The Netherlands

**Keywords:** Self-control, Internalizing, Externalizing, Family functioning, Random-intercept cross-lagged panel model, Adolescents

## Abstract

Adolescents’ self-control develops in the context of mental health and family functioning, but it is unclear how the interplay of self-control, mental health, and family functioning unfolds across time within individuals. Separating within-person from between-person effects, random-intercept cross-lagged panel models were applied to adolescents (from ages 11 to 26) from a Dutch cohort (*n* = 2228, 51% female). Adolescents with low self-control were likely to have mental health problems and poorly functioning families. Although within-person changes in the study variables were not meaningfully associated in a reciprocal manner, changes in self-control and mental health were concurrently associated. This suggests that besides stable connections between self-control, mental health, and family functioning in adolescence and young adulthood, changes in self-control and mental health are developmentally linked as well.

## Introduction

Adequate self-control in early life is associated with a vast range of positive long-term outcomes, including physical and mental health, higher education levels, better career opportunities, and financial security (Duckworth, 2011). In turn, low self-control has been linked to problems in school or work functioning, experiences of psychological distress, and the development of mental health problems (Nedelec & Beaver, 2014). Understanding self-control development in youth is crucial not only for improving individuals’ quality of life, but also for reducing social costs related to, for example, health care needs caused by low self-control (Nedelec & Beaver, 2014). According to the transactional model (Sameroff, 2009, Sameroff & MacKenzie, 2003), self-control development occurs through interactions between individuals and their families. These interactions are assumed to occur *within* persons and families: changes in adolescents’ family environment can result in changes in adolescent self-control and mental health problems, and vice versa, changes in adolescent self-control and mental health can lead to changes in their family environment. However, most empirical findings on associations between self-control, mental health, and family factors are based on between-person approaches such as group-based regression or cross-lagged models. Thus, previous research has rarely examined whether changes in self-control, mental health problems, and family factors actually predict each other across time within adolescents and their families. The current study aims to fill these gaps by assessing within-person reciprocal associations between self-control, mental health problems, and family functioning among 2228 Dutch adolescents who were followed from the age of 11 to 26.

### Self-control in Adolescents and Young Adults

Self-control is defined as the capacity to engage in voluntary actions to pursue valued distal goals over conflicting proximal urges (Duckworth & Steinberg, 2015, Nigg, 2017). Self-control enables adolescents to inhibit undesired actions, emotions, and cognitions and to strengthen desired ones for the sake of long-term adaptation. Major cognitive, emotional, and social developments occur during adolescence, and these developments continue up until young adulthood (Hazen et al., 2008). Self-control can help adolescents and young adults to successfully adjust to these changes: individuals with high self-control are likely to develop fewer mental health problems and obtain higher education levels and socioeconomic status than individuals with low self-control (de Ridder et al., 2012, Fergusson et al., 2013).

While self-control increases most rapidly during the first decade of life, it continues to develop—albeit at a slower pace—during subsequent phases of life (Vazsonyi & Jiskrova, 2018). On average, self-control matures or increases across adolescence and young adulthood (Forrest et al., 2019; Shulman et al., 2015; Winfree et al., 2006; Zondervan-Zwijnenburg et al., 2020), but there is substantial heterogeneity in the development of self-control, reflected in diverse trajectories of self-control development in adolescents and young adults (Burt et al., 2014; Forrest et al., 2019). This suggests that self-control is not stable during adolescence and young adulthood and that its development is probably not predetermined. That raises the question of which factors drive changes in self-control during this period. To answer this question, the current study focuses on two factors that may affect and be affected by self-control, namely mental health problems and family functioning.

### Mental Health Problems and Self-control in Youth

Mental health problems, which are commonly divided into externalizing problems (e.g., aggressive and rule-breaking behaviors) and internalizing problems (e.g., anxious and depressed behaviors) (McDermott et al., 2017), often emerge during youth (Belfer, 2008; Kessler et al., 2007). This is partly due to the increased plasticity in regions of the cortex, which not only facilitates development but also increases adolescents’ sensitivity to the effects of stress on mental health (Fuhrmann et al., 2015). Mental health problems have been related to structural and functional abnormalities in the prefrontal cortex that can cause impairments in executive functions (Snyder, 2013) such as working memory, shifting, and inhibition. These executive functions support self-control by helping adolescents to suppress undesired urges and facilitating goal-directed actions (Duckworth & Steinberg, 2015). Thus, mental health problems may lead to low self-control through impaired executive functions. Mental health problems not only affect self-control but can be affected by self-control as well. When adolescents with low self-control fail in achieving goals, they may develop a sense of low self-efficacy in school or work functioning, which is an established risk factor for depressive and anxiety symptoms (Bandura, 1989). Furthermore, self-control helps to inhibit aggressive behaviors (Denson et al., 2012). This suggests that self-control can be a protective factor of both internalizing and externalizing problems in youth.

The interplay between self-control and mental health problems in youth can, to some extent, be captured by studying reciprocal or cross-lagged effects between self-control and mental health problems across time. Studies on externalizing problems in adolescents have found reciprocal associations between externalizing problems and measures related to self-control, such as effortful control (Esposito et al., 2017; Paige et al., 2021; but see Eisenberg et al., 2005). Evidence regarding the directionality of associations between self-control and internalizing problems is rather mixed (Donati et al., 2021; McLaughlin et al., 2011; Morea & Calvete, 2021; Situ et al., 2021). For example, reciprocal associations between self-control and internalizing problems were found in a study that followed college freshmen for six months (Situ et al., 2021). A study examining cross-lagged paths between depressive symptoms and executive functions (cognitive flexibility and selective attention) in adolescents between 12 and 17 years old only found unidirectional effects from depressive symptoms to executive functioning (Morea & Calvete, 2021), while another study found unidirectional effects from emotion dysregulation to internalizing problems in adolescents (McLaughlin et al., 2011). Thus, it is not clear whether self-control and internalizing problems are reciprocally associated.

### Family Functioning and Self-control in Youth

Family functioning is another potentially important factor in adolescents’ self-control development (Cecil et al., 2012; Holmes et al., 2019; Richards et al., 2019). Family functioning reflects how the family operates as a whole, including interactions, relationships, conflicts, and cohesion among family members, and represents the organization, adaptability, and quality of communication within a family (Lewandowski, A. S., Palermo et al., 2010). The current study focuses on family functioning based on the McMaster Model, encompassing family problem-solving, communication, roles, affective responsiveness, behavior control, and general functioning (Epstein et al., 1983). Family functioning can shape the behavior of family members and vice versa (Miller, Ryan, Keitner, Bishop, & Epstein, 2000). More specifically, the family environment can help adolescents to control undesirable urges by positive and constructive ways of communication and conflict resolution that can serve as example behaviors, and it can provide a supportive environment to practice these behaviors (Peterson, 2005). In turn, adolescents who can effectively regulate negative emotions and undesired behaviors are likely to build positive relationships with other family members and are unlikely to provoke conflicts. Hence, bidirectional influences between family functioning and adolescents’ self-control are conceivable.

Numerous studies have focused on the associations between family factors (e.g., parenting, parent–child relationships, and family functioning) and self-control in children and adolescents, and the findings suggest that family environment plays an important role in self-control development (see reviews by Farley & Kim-Spoon, 2014; Kiss et al., 2014; Scully et al., 2020). Yet, not many studies have tested transactional associations between family factors and self-control. The ones that did focused on childhood and early adolescence and largely ignored late adolescence and young adulthood, life phases that are also highly relevant for understanding the development of self-control. According to these studies, parenting not only predicts children’s self-control, but also the other way around (Cecil et al., 2012; Eisenberg et al., 2005; Tiberio et al., 2016; but see Neppl et al., 2020). Tiberio and colleagues followed children from age 3 to 14 years and found that effortful control influenced later parenting during childhood while parenting had effects on subsequent self-control in early adolescence. This suggests that developmental periods might matter when it comes to examining the direction of effects between family factors and self-control, but this has not been systematically investigated using data across adolescence and young adulthood. Furthermore, previous research on reciprocal associations between self-control and family environment focused solely on parenting behaviors. Although parenting is an important component of family functioning, family functioning reflects a broader range of family factors, including relationships with siblings and relationships among individuals other than the target child. Altogether, there is a scarcity of knowledge regarding reciprocal associations between self-control and family functioning across the whole period of adolescence and young adulthood.

### Self-control, Mental Health, and Family Functioning in Youth

As discussed above, mental health problems and family functioning may affect and be affected by self-control. In addition to this, families that do not function well can increase the risk of adolescents’ mental health problems, and adolescents’ mental health issues can stress the family and hence deteriorate its functioning (e.g., Delsing et al., 2005; Serbin et al., 2015). Therefore, these three factors are interrelated and could best be investigated in concert to assess how they affect one another.

Apart from allowing to estimate effects that are adjusted for each other, modeling self-control, mental health problems, and family functioning together offers the advantage of elucidating pathways between the three. For instance, self-control might be involved in associations between family functioning and youth mental health problems as a mediator. Previous studies on this topic focused on externalizing problems in particular and suggest that self-control partly mediates effects of family factors on externalizing problems, with negative family factors predicting poor self-control, and poor self-control predicting externalizing problems (Bradley & Corwyn, 2013; Eisenberg et al., 2005; Eisenberg et al., 2015; Janssen et al., 2016; Sulik et al., 2015; but see Van Heel et al., 2020). Moreover, Eisenberg et al. (2015) suggested that self-control (measured by effortful control in their study) could mediate the effect of mental health problems on parenting in young children.

The above studies did not explore the many other possible pathways between adolescents’ self-control, mental health, and family functioning, such as self-control affecting family functioning and family functioning in turn affecting mental health problems (as suggested by Elam et al., 2016). One exception was the study of Eisenberg et al. (2015) that examined the cross-lagged paths among effortful control, parenting, and externalizing problems in young children. Using cross-lagged panel models with three measurement waves, at 30, 42, and 54 months of age, they found that effortful control was reciprocally associated with parenting and externalizing problems but parenting and externalizing problems were not reciprocally related to one another. Another exception was the study on maltreatment, self-control, and aggression in early adolescence (Yang et al., 2021). They followed up young adolescents for 2.5 years, with 6-months intervals, and found the reciprocal associations among the study variables, using a cross-lagged panel model. As far as the authors are aware, these are thus far the only studies that tested reciprocal associations among self-control, mental health, and aspects of the family environment.

In sum, previous studies have mostly focused on examining the mediation of self-control in the associations between family functioning and externalizing problems, ignoring internalizing problems. Moreover, except for the two studies noted above, they did not examine how self-control, mental health problems, and family functioning affect each other—not limited to certain mediation paths. Thus, the understanding of the reciprocal associations among these factors in adolescence and young adulthood remains limited. This is regrettable from both a scientific and a public health perspective because spiral effects, in which the three factors or a subset of these exert a negative—or positive—influence on each other over time, can offer clues about which factor may be the most effective target for prevention or intervention strategies. Altogether, this calls for more research to unravel whether and how self-control, mental health problems, and family functioning are related to each other across time.

### Between- and Within-person Dynamics

In previous research on reciprocal associations between self-control, mental health problems, and family functioning, these relations were usually tested based on a mixture of between- and within-individual variance. Cross-lagged panel models (CLPMs) are one of the most commonly used approaches to test reciprocal associations between study constructs. By using multiple measurement waves, CLPMs test contemporaneous, autoregressive, and cross-lagged—reciprocal—associations during a certain developmental period. Such tests do not provide a perfect match with the research questions, because the transactional effects between self-control, mental health, and family functioning are, like all developmental processes, theorized to take place within individuals (or families). Cross-lagged paths from a CLPM, for example, do not tell whether adolescents who experienced worse family functioning than usual at a particular time point, report a subsequent decrease in self-control (Orth et al., 2021).

The random-intercept cross-lagged panel model (RI-CLPM) is a viable option to investigate the transactional associations between self-control, mental health problems, and family functioning at the within-person level (Hamaker et al., 2015). The RI-CLPM distinguishes within- from between-individual associations by adding random intercepts to the traditional CLPM. In the RI-CLPM, within-person associations are no longer contaminated by between-person differences (Hamaker et al., 2015), hence the CLPM and RI-CLPM could yield different results for the same data. For example, in a study of adolescent mental health problems and family functioning, mental health problems were negatively associated with subsequent family functioning when a CLPM was used, but no cross-lagged paths were found employing a RI-CLPM (Mastrotheodoros et al., 2020). Thus, in order to test whether cross-lagged paths occur within individuals, it is necessary to distinguish within-person effects from between-person stable differences.

Purely within-person transactional associations between self-control, mental health problems, and family functioning remain largely unexplored thus far. More and more studies have tested within-person transactional associations, but they have mostly examined only a part of the interplay among self-control, mental health, and family factors: within-person effects between mental health problems and family factors, such as family functioning, family cohesion, and parent–child conflict (Fredrick et al., 2021; Lougheed et al., 2020; Mastrotheodoros et al., 2020), between self-control measures and family factors (Neppl et al., 2020), and between mental health problems and self-control measures (Maasalo et al., 2021) in children and adolescents. Using RI-CLPM to distinguish within-person from between-person effects, these studies found between-person associations among the variables under study, but no longitudinal within-person associations. This seems to indicate that within-person cross-lagged effects between self-control, mental health problems, and family functioning are harder to detect than effects based on a mixture of between- and within-person variance, but the evidence is still very limited.

## Current Study

The review above indicates a gap in the research on self-control and its interplay with mental health and family functioning. Despite the plausibility of developmental transactions among self-control, mental health, and family functioning, this transactional process has not yet been studied in adolescents and young adults. The present study aimed to fill that gap by using data from repeated assessments of self-control, internalizing and externalizing problems, and family functioning throughout adolescence up until young adulthood, that is, from ages 11 to 26 years. A transactional view on self-control development implies that it occurs within individuals. Hence, an analytical approach that distinguishes between-individual from within-individual effects is employed.

## Methods

### Participants and Procedure

Data came from the TRacking Adolescents’ Individual Lives Survey (TRAILS), which consists of a population-based and a clinically referred (ever) cohort, recruited in the North of the Netherlands. The TRAILS participants were aged ten to twelve years at the first wave of the survey. The first wave of data collection ran from March 2001 to July 2002 for the population-based cohort and from September 2004 to December 2005 for the clinically referred cohort. In total, 3145 and 1264 children were approached for the population and clinical cohort, respectively, of whom 2230 (76.0%) and 543 (43.0%) agreed to participate. Most of them were Dutch (86.5% and 98.1% for population and clinical cohort, respectively) and from dual-parent families (97.6% and 97.8% for population and clinical cohort, respectively). Percentages of participants who had a parent with a low educational level were 32.6% for the population cohort and 48.3% for the clinical cohort. Follow-up assessments were conducted every two or three years, resulting in six measurement waves (T_1_ to T_6_) for the population-based cohort, with response rates ranging from 72.6% to 96.4%. Mean ages of the TRAILS population cohort were 11.06 years at T_1_ (*SD* = 0.55), 13.57 at T_2_ (*SD* = 0.53), 16.28 at T_3_ (*SD* = 0.71), 19.08 at T_4_ (*SD* = 0.60), 22.29 at T_5_ (*SD* = 0.65), and 25.66 at T_6_ (*SD* = 0.60). The clinical cohort was assessed five times (T_1_ to T_5_), with response rates ranging between 74.2% and 85.1%. The mean age of the clinical cohort corresponded to the population cohort, ranging from 11.11 years at T_1_ (*SD* = 0.50) to 21.96 at T_5_ (*SD* = 0.74). Extensive information regarding recruitment and assessment procedures can be found on the TRAILS website (https://www.trails.nl/en/) and in the cohort profile (Huisman et al., 2008). After excluding two participants who had missing values for the study variables across all measurement waves, a total of 2228 participants (51% female) from the population cohort and 543 participants (34% female) from the clinical cohort were included in the current study.

### Measures

#### Self-control problems

Self-control problems were assessed by the ASCS (Achenbach System of Empirically Based Assessment Self-Control Scale) (Willems et al., 2018). For T_1_ to T_3_, the items came from the Youth Self-Report (YSR) (Achenbach, 1991), for T_4_ to T_6_ from the Adult Self-Report (ASR) (Achenbach & Rescorla, 2003). The YSR and ASR assess the presence of behavioral and emotional problems in the past six months. Participants rated the items as 0 (not true), 1 (somewhat or sometimes true), or 2 (very or often true). A higher score indicates more self-control problems. The original ASCS consists of eight items, measuring attention problems, aggressive behavior, and rule-breaking behavior. The item ‘Inattentive or easily distracted’ was not included in the ASR and hence excluded, leaving seven items (Table S1) with satisfactory internal consistency across the study waves (Cronbach’s αs = 0.66–0.74; Table 1).

**Table 1:** Means, standard deviations, and internal consistency coefficients α for all study variables at each measurement wave of the TRAILS population-based and clinically-referred cohorts

	Population cohort		Clinical cohort		
Variable	M (*SD*)	Cronbach’s α	M (*SD*)	Cronbach’s α	*p* ^a^
Self-control problems T_1_	0.48 (0.32)	0.66	0.60 (0.37)	0.69	<0.001
Self-control problems T_2_	0.56 (0.34)	0.68	0.65 (0.36)	0.69	<0.001
Self-control problems T_3_	0.57 (0.34)	0.70	0.71 (0.36)	0.67	<0.001
Self-control problems T_4_	0.44 (0.35)	0.72	0.59 (0.39)	0.73	<0.001
Self-control problems T_5_	0.40 (0.32)	0.69	0.52 (0.36)	0.74	<0.001
Self-control problems T_6_	0.42 (0.34)	0.71	–	–	
Family functioning T_1_	1.77 (0.36)	0.85	1.93 (0.41)	0.87	<0.001
Family functioning T_2_	1.64 (0.40)	0.87	1.86 (0.41)	0.88	<0.001
Family functioning T_3_	1.65 (0.40)	0.87	1.75 (0.46)	0.90	<0.001
Family functioning T_4_	1.68 (0.39)	0.87	1.80 (0.46)	0.89	<0.001
Family functioning T_5_	1.65 (0.41)	0.88	1.74 (0.47)	0.90	0.001
Family functioning T_6_	1.71 (0.40)	0.88	–	–	
Internalizing problems T_1_	0.36 (0.24)	0.86	0.40 (0.25)	0.85	0.001
Internalizing problems T_2_	0.34 (0.25)	0.88	0.38 (0.26)	0.86	<0.001
Internalizing problems T_3_	0.32 (0.25)	0.88	0.34 (0.25)	0.87	0.169
Internalizing problems T_4_	0.26 (0.25)	0.90	0.35 (0.30)	0.90	<0.001
Internalizing problems T_5_	0.28 (0.26)	0.90	0.36 (0.28)	0.89	<0.001
Internalizing problems T_6_	0.34 (0.29)	0.91	–	–	
Externalizing problems T_1_	0.26 (0.19)	0.77	0.32 (0.23)	0.79	<0.001
Externalizing problems T_2_	0.25 (0.19)	0.77	0.30 (0.22)	0.78	<0.001
Externalizing problems T_3_	0.27 (0.21)	0.79	0.32 (0.23)	0.81	<0.001
Externalizing problems T_4_	0.17 (0.17)	0.76	0.22 (0.20)	0.74	<0.001
Externalizing problems T_5_	0.14 (0.14)	0.68	0.18 (0.16)	0.70	<0.001
Externalizing problems T_6_	0.12 (0.13)	0.67	–	–	

^a^*p*-value of independent-samples *t*-test

#### Mental health problems

Self-reported internalizing and externalizing problem scores were assessed with the YSR (T_1_ to T_3_) and ASR (T_4_ to T_6_), with ratings as described above. The internalizing problems scale covers anxious/depressed behaviors, withdrawn/depressed behaviors, and somatic complaints; the externalizing problems scale aggressive and rule-breaking behaviors. Items were included only when they had the same meaning in the YSR and the ASR. Four items were excluded from the externalizing problems scale because they are included in the ASCS (see Table S1). In total, 28 and 20 items were used to measure internalizing (αs = 0.85–0.91) and externalizing (αs = 0.67–0.81) problems respectively (Table 1).

#### Family functioning

Family functioning was reported by one of the parents (usually the mother) of the TRAILS participants by means of a modified version of the McMaster Family Assessment Device (FAD) (Epstein et al., 1983). The scale contains 12 items (αs = 0.85–0.90; Table 1) covering six dimensions of family functioning: problem-solving, communication, roles, affective responsiveness, behavior control, and general functioning. The parents rated the items on a 4-point Likert scale ranging from 1 (totally disagree) to 4 (totally agree). Positively worded items were reverse coded so that a higher score indicates worse family functioning.

### Statistical Analyses

The analytic plan for this study has been preregistered on the Open Science Framework (https://osf.io/7qwk9/). First, an a priori Monte Carlo simulation was conducted to determine if the sample size was large enough to use the random-intercept cross-lagged panel models (RI-CLPMs) to detect the smallest effect size of interest of 0.10. Details on the Monte Carlo simulation are provided in the Supplementary Information. The Intraclass Correlation Coefficients (ICCs) of the study variables were then calculated to check the relative amount of between-person and within-person variance. The RI-CLPMs deviated from the pre-registered analysis plan. Mean scores were used for the models because reliable estimations were not obtained from multiple indicator and factor score RI-CLPMs (see Supplementary Information on attempted multiple indicator and factor score RI-CLPMs). First, separate models were fit for internalizing and externalizing problems using data from the TRAILS population cohort (abbreviated as PC from this point on) from T_1_ to T_6_. To capture stable between-person differences, random intercepts were estimated using three overarching between-person latent factors (one for self-control, one for externalizing or internalizing problems, and one for family functioning) across all measurement waves. To assess within-person effects, within-person latent variables were regressed for each study variable at each wave with loadings constrained to 1. Three types of within-person paths were specified: stability paths (auto-regressive paths) of the same latent factors across time, within-wave associations between the latent variables (concurrent paths), and cross-lagged effects (paths between latent variables over time). The measurement error variance was constrained to 0, which assumes that the variation in the observed scores was captured by the within- and between-person latent factor structure (Mulder & Hamaker, 2021). Maximum likelihood estimations with robust standard errors were applied for the RI-CLPMs.

To achieve the most parsimonious RI-CLPMs, an initial baseline RI-CLPM where all paths were free to vary over time was compared with models in which within-person paths were constrained to be equal over time, one-by-one in the order of stability, cross-lagged, and concurrent effects. After constraining each path, the model fit was compared using the Satorra–Bentler scaled chi-square difference test (Satorra & Bentler, 2001), and Root Mean Square Error of Approximation (RMSEA), Bentler’s Comparative Fit Index (CFI), and Standardized Root Mean Square Residual (SRMR) were used to assess model fit. The fit was considered acceptable if the upper boundary of the 90% confidence interval of RMSEA was ≤ 0.10, CFI was ≥ 0.95, and SRMR was ≤ 0.08 (Kline, 2016). If the model fit became significantly worse after constraining a path, that path was allowed to vary over time. A model with the maximum number of constrained within-person paths was considered a parsimonious model since it has the minimum number of parameters to compute (see Tables S3 and S4 for model fitting).

To examine whether the results were robust across populations, the same procedure was repeated with the data of the TRAILS clinical cohort (abbreviated as CC) from T_1_ to T_5_. In addition, traditional CLPMs were examined using the PC, and the fit was compared to the PC RI-CLPMs to test whether the separation of within- and between-person variances in a RI-CLPM explained the data better than a CLPM with aggregated variances. Details of the sensitivity analyses can be found in the Supplementary Information.

SPSS version 26 was used to calculate ICCs and mean scores for self-control, mental health problems, and family functioning. The rest of the statistical analysis was performed with Mplus Version 8.0, with a smallest effect size of interest of 0.10 and a significance level of 0.05 without adjustment for multiple testing.

## Results

### Descriptive Statistics

Table 1 provides an overview of the means, standard deviations, Cronbach’s αs, and *p*-values from independent sample t-tests of the study variables in the PC and CC. The correlations among self-control, mental health problems, and family functioning in the PC are shown in Table 2. The correlations among the study variables in the CC were largely similar to those from the PC, except for those between self-control and family functioning, which were slightly lower than found in the PC (*r*s = −0.09 to 0.09, Table S2). Across the study waves, the ICCs of self-control, internalizing problems, externalizing problems, and family functioning were 0.40, 0.45, 0.37, and 0.46, respectively, in the PC, and 0.36, 0.39, 0.34, and 0.49 in the CC. This indicates that, overall, most of the variance was due to within-person fluctuations.

**Table 2:** Correlations between self-control, mental health problems, and family functioning in the population cohort

1.	1	2	3	4	5	6	7	8	9	10	11	12	13	14	15	16	17	18	19	20	21	22	23
1. SC T1	–																						
2. SC T2	0.41	–																					
3. SC T3	0.33	0.51	–																				
4. SC T4	0.28	0.40	0.54	–																			
5. SC T5	0.25	0.35	0.48	0.57	–																		
6. SC T6	0.25	0.30	0.41	0.56	0.61	–																	
7. FF T1	0.09	0.08	0.09	0.08	0.07	0.07	–																
8. FF T2	0.08	0.12	0.11	0.13	0.10	0.11	0.51	–															
9. FF T3	0.07	0.10	0.15	0.14	0.12	0.09	0.45	0.52	–														
10. FF T4	0.05	0.08	0.13	0.12	0.11	0.11	0.42	0.46	0.55	–													
11. FF T5	0.06	0.11	0.11	0.11	0.12	0.11	0.37	0.40	0.48	0.57	–												
12. FF T6	0.07	0.07	0.10	0.08	0.10	0.14	0.34	0.37	0.39	0.48	0.56	–											
13. INT T1	0.56	0.32	0.27	0.23	0.21	0.21	0.10	0.09	0.06	0.10	0.08	0.11	–										
14. INT T2	0.27	0.54	0.34	0.31	0.32	0.26	0.09	0.12	0.11	0.09	0.10	0.07	0.50	–									
15. INT T3	0.19	0.34	0.52	0.37	0.38	0.34	0.10	0.13	0.15	0.13	0.15	0.15	0.39	0.58	–								
16. INT T4	0.21	0.29	0.36	0.60	0.41	0.44	0.07	0.14	0.15	0.15	0.12	0.12	0.33	0.45	0.59	–							
17. INT T5	0.17	0.25	0.34	0.37	0.62	0.45	0.08	0.11	0.12	0.12	0.15	0.14	0.28	0.41	0.55	0.59	–						
18. INT T6	0.17	0.21	0.31	0.38	0.43	0.65	0.08	0.10	0.12	0.11	0.14	0.16	0.27	0.38	0.51	0.56	0.66	–					
19. EXT T1	0.63	0.31	0.21	0.21	0.18	0.20	0.11	0.10	0.06	0.05^a^	0.07	0.09	0.48	0.19	0.10	0.14	0.09	0.09	–				
20. EXT T2	0.32	0.60	0.37	0.30	0.24	0.23	0.11	0.15	0.07	0.09	0.09	0.09	0.26	0.37	0.21	0.18	0.13	0.12	0.42	–			
21. EXT T3	0.23	0.36	0.60	0.39	0.33	0.31	0.09	0.10	0.14	0.10	0.10	0.09	0.14	0.18	0.29	0.18	0.17	0.13	0.30	0.50	–		
22. EXT T4	0.24	0.30	0.41	0.65	0.39	0.44	0.04^a^	0.10	0.11	0.12	0.11	0.09	0.18	0.18	0.23	0.41	0.22	0.27	0.28	0.38	0.49	–	
23. EXT T5	0.19	0.26	0.35	0.40	0.61	0.45	0.08	0.11	0.12	0.12	0.11	0.15	0.13	0.17	0.20	0.26	0.43	0.30	0.22	0.29	0.43	0.52	–
24. EXT T6	0.21	0.25	0.32	0.44	0.41	0.64	0.05^a^	0.09	0.09	0.09	0.10	0.14	0.18	0.17	0.21	0.32	0.29	0.46	0.22	0.29	0.39	0.54	0.56

*Note*. SC = self-reported self-control problems; FF = parent-reported family functioning; INT = self-reported internalizing problems; EXT = self-reported externalizing problems

^a^Correlation coefficients with a *p*-value of > 0.05

### RI-CLPMs in the Population Cohort

Both parsimonious RI-CLPMs for internalizing and externalizing problems showed a good fit to the PC data (internalizing problems RI-CLPM: RMSEA = 0.025, CFI = 0.985, SRMR = 0.041; externalizing problems RI-CLPM: RMSEA = 0.027, CFI = 0.982, SRMR = 0.042). Details of the PC RI-CLPMs can be found in the Supporting Information, Tables S3 and S4.

**Fig. 1:**
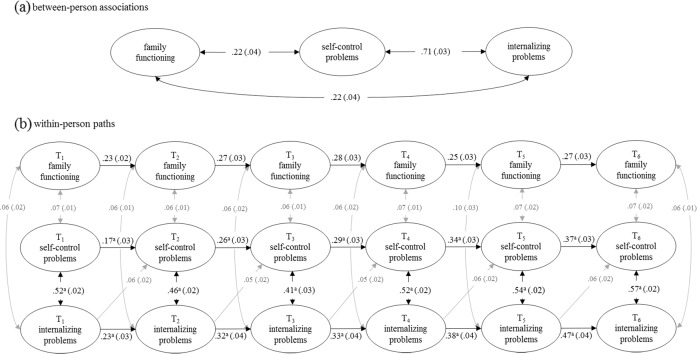
RI-CLPM standardized estimates (standard errors) for self-reported self-control problems, self-reported internalizing problems, and parent-reported family functioning in the population cohort. Only statistically significant (*p* < 0.05) paths are visualized in this figure. Gray lines depict paths with standardized estimates < 0.10. ^a^paths that are not constrained across time

**Fig. 2:**
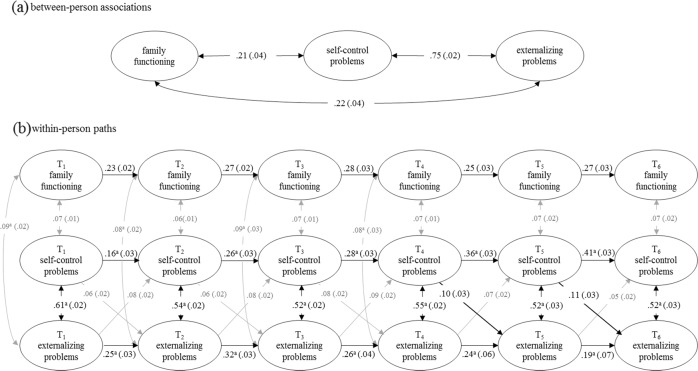
RI-CLPM standardized estimates (standard errors) for self-reported self-control problems, self-reported externalizing problems, and parent-reported family functioning in the population cohort. Only statistically significant (*p* < 0.05) paths are visualized in this figure. Gray lines depict the paths with the standardized estimates < 0.10. ^a^ paths that are not constrained across time

At the between-person level (Figs. 1a and 2a), participants with more self-control problems had more externalizing (*β* = 0.75) and internalizing problems (*β* = 0.71), and worse family functioning reported by parents (*β* = 0.21 from the externalizing problems RI-CLPM; *β* = 0.22 from the internalizing problems RI-CLPM). Similarly, having more mental health problems was associated with worse family functioning (*β* = 0.22 in both models).

At the within-person level, self-control and mental health problems had moderate concurrent associations (*β*s = 0.41 to 0.57 for internalizing problems and 0.52 to 0.61 for externalizing problems). All other concurrent associations were weak (*β*s ≤ 0.10) but significant, except for nonsignificant concurrent associations between family functioning and externalizing problems at T_5_ and T_6_. All variables showed weak to moderate stability (*β*s = 0.16 to 0.47). Whereas the stability of self-control and internalizing problems increased monotonously as participants grew older, the stability of externalizing problems decreased from early adolescence (T_2_) onwards. Weak cross-lagged paths were found from self-control to externalizing problems (*β*s = 0.06 to 0.11) and from mental health problems to self-control with the effect sizes smaller than 0.10 (*β*s = 0.05 to 0.09) across the six measurement waves. There were no statically significant cross-lagged paths between self-control and family functioning, nor between family functioning and mental health problems. All path coefficients are reported in Table S5.

### RI-CLPMs in the Clinical Cohort

In the CC, self-control and mental health problems were associated at the between-person level (*β* = 0.38 and 0.82 for internalizing and externalizing problems, respectively), but family functioning was not significantly associated with either self-control or mental health problems at the between-person level. At the within-person level, significant paths and their estimated effect sizes were, in general, similar to those from the PC, although there were some differences between the cohorts. The stability paths of self-control and externalizing problems were constrained across the measurement waves in the CC, while unconstrained in the PC. Nevertheless, the effect sizes were similar. Further, the concurrent paths between family functioning and mental health problems were not significant in the CC, while these effects were trivial but significant in the PC. The path coefficients from the CC RI-CLPMs are reported in Table S6.

### CLPMs

The PC CLPMs showed a significantly worse fit than the respective RI-CLPMs (Table S7). All CLPM stability paths had larger effect sizes (*β*s = 0.35 to 0.64) than the within-person stability effects in the RI-CLPMs (*β*s = 0.16 to 0.47). Similar to the RI-CLPMs, the CLPMs indicated a monotonous increase in the stability of self-control and internalizing problems. The concurrent paths in the CLPMs showed the same patterns as the RI-CLPMs, with slightly stronger effects. Small but significant effects were found for the cross-lagged paths from mental health problems to self-control (*β*s = 0.07 to 0.12) and from self-control to externalizing problems (*β*s = 0.09 to 0.14). The other cross-lagged paths had trivial effect sizes, which was consistent with the findings from the RI-CLPMs. The standardized path coefficients of the CLPMs are presented in Table S8.

### Post Hoc Analyses: RI-CLPMs with Different Informants

The between-person correlations and within-person concurrent associations of self-reported self-control and mental health problems were much stronger than the associations of self-reported self-control or mental health problems with parent-reported family functioning. This might have been due to shared method variance, as self-control and mental health problems were measured by the same informant. To explore the effect of mono-informant bias on the findings, post hoc RI-CLPMs were conducted using self- and parent-reported self-control problems and self-, parent-, and teacher-reported mental health problems in the PC. Because only parents reported family functioning, other informants for family functioning were not available. Parent- and teacher-reported items were not assessed after T_3._ Therefore, post hoc RI-CLPMs were conducted across T_1_ to T_3_ (for details on measures and procedure, see Supplementary Information).

The results from the post hoc analyses suggest that the associations found between self-reported self-control and mental health problems were inflated due to the shared method variance: the between-and within-person concurrent associations between self-control and mental health problems were much higher (two- to four-fold) when reported by the same informant than when reported by different informants. Other associations in the post hoc analyses also had stronger effect sizes when the variables were reported from the same informant. For example, the between-person associations of parent-reported family functioning with parent-reported self-control problems were stronger (*β*s = 0.36 to 0.38) than those between parent-reported family functioning and self-reported self-control problems (*β*s = 0.12 to 0.15). Likewise, within-person concurrent paths among the variables had, in general, stronger effect sizes when the variables were reported by the same informant than when they were reported by different informants (Tables S9 and S10). Finally, the results from post hoc analyses suggest that when accounting for shared-method variance due to shared informants, the associations between self-control and mental health problems were still stronger than the associations between each of these variables and family functioning.

## Discussion

Adolescence is a period involving major cognitive, emotional, and social developments. Adequate adjustment to these changes can be a challenging task for both adolescents themselves and their direct environment. Self-control is an important ability that further develops in adolescence through an interplay between adolescents and their families. This interplay can be referred to as a transactional process; according to the transactional development model, changes in an individual and his or her environment influence each other across time (Sameroff, 2009; Sameroff & MacKenzie, 2003). In the current study, mental health problems and family functioning were investigated as potential factors involved in the transactional development of self-control. An important issue in studying the transactional development of self-control is that the ecological level of analysis should match the research questions. Transactional associations between self-control, mental health problems, and family functioning occur at a within-person level. Yet, the within-person transactional development of self-control in relation to mental health problems and family functioning across adolescence and young adulthood had not been explored. The current study addressed this gap in the literature by examining between- and within-person associations among self-control problems, internalizing and externalizing problems, and family functioning between the ages of 11 to 26 years.

Substantial between-person associations were found, suggesting that low self-control, more mental health problems and low family functioning tend to co-occur across adolescence and young adulthood. At the within-person level, no meaningful cross-lagged (transactional) effects were found among self-control, mental health problems, and family functioning. While significant positive within-person cross-lagged effects were found from internalizing to self-control problems, as well as positive reciprocal effects between externalizing and self-control problems, most effect sizes were smaller than the predefined smallest effect size of interest of 0.10; hence, they were not considered meaningful. Nevertheless, self-control and mental health problems were concurrently associated at the within-person level, across adolescence and young adulthood. That is, adolescents reporting more self-control problems relative to their average level tended to report more mental health problems during the same wave.

### Within-person Development of Self-control, Mental Health Problems, and Family Functioning

Although it is theoretically plausible that self-control has transactional associations with mental health problems and family functioning at the within-person level, the current study did not find empirical evidence supporting within-person cross-lagged effects in adolescents and young adults. The lack of robust within-person reciprocal effects in youth is concordant with other RI-CLPM studies. Yang et al. (2021) examined within-person cross-lagged effects among self-control, aggression, and maltreatment, in early adolescents who were 10.4 years at the baseline and were followed up every six months for 2.5 years. They found some significant within-person cross-lagged effects among the study variables, but most of the effects were small (standardized path coefficients < 0.1) except for the within-person effects of aggression on maltreatment and self-control during the first and second study waves. Furthermore, it is difficult to compare their findings with ours since they studied a specific and severe type of family malfunctioning, i.e., child maltreatment, whereas broader family functioning was measured in the current study. Mastrotheodoros et al. (2020) and Fredrick and colleagues (2021) found no significant cross-lagged associations between family factors (family functioning and family cohesion) and mental health problems (depressive symptoms, anxiety, and anger) at the within-person level in samples of adolescents, which is comparable to the results of the current study. The findings of the current study are further in line with other research in which changes in self-control measures were not associated with subsequent changes in family environment or mental health problems in mid-childhood, nor the other way around, at the within-person level (Maasalo et al., 2021; Neppl et al., 2020).

The fact that no substantial within-person cross-lagged effects were found in the current or previous studies might be related to the rather long intervals between the study waves. In the current study, cross-lagged associations were examined with time intervals of 2.5–3 years, and previous RI-CLPM studies were conducted with time lags of 6–12 months. Causal interactions between individual and family factors might occur on a shorter time scale, for instance within weeks or days. If so, the within-person concurrent associations found between self-control and mental health problems may reflect effects occurring at shorter intervals within individuals. Related to this, and given that Mastrotheodoros et al. (2020) did not find within-person reciprocal associations using intervals of six months, the time frame of the YSR and ASR (measuring problems in the past 6 months) might not be optimal to capture within-person dynamics in adolescents. If the most relevant interactions between adolescents’ behavior and family functioning occur on a daily or weekly level, a conventional cohort study design may not be able to capture the within individual transactional development, and diary studies might be more suitable to assess the interactions between individual and family problems. As such, it may be that the concurrent associations found between self-control and mental health problems in the current study may (in part) reflect within individual transactional effects. The weak concurrent associations of family functioning with self-control and mental health problems might indicate that family functioning does not have strong transactional effects on self-control and mental health problems in adolescents and young adults. Given the limitations of the data, definitive conclusions cannot be drawn regarding the within individual effects among self-control, mental health, and family functioning from adolescence to young adulthood. Studies utilizing data collected with short time intervals between measures are warranted to shed more light on this issue.

### Between-person Relations among Self-control, Mental Health Problems, and Family Functioning

Stable between-person associations between self-control, mental health problems, and family functioning were found across adolescence and young adulthood in the population cohort. To some extent, an accumulation of effects within individuals, possibly occurring at short-term intervals, could have resulted in heightened self-control and mental health problems and worse family functioning in some adolescents compared to others. In addition, shared predictors such as socioeconomic status and genetic predisposition may partly explain the found between-person associations (Conger et al., 2010). According to the social causation hypothesis, socioeconomic disadvantage in a family can lead to both individual- and family-related problems (Botha et al., 2018; Wadsworth & Achenbach, 2005). From a social selection perspective, genetic predispositions to psychopathology can lead to differences in both family functioning and developmental problems (McLeod & Kaiser, 2004).

Genetic underpinnings may partly explain the differences in findings from clinical and nonclinical (population) cohorts. That is, in the clinical cohort, unlike the findings from the population cohort, family functioning was not associated with either self-control or mental health problems at the between-person level. A possible explanation for the lack of association in the clinical cohort could be that adolescents from the population and clinical cohort potentially have different genetic predispositions in developing mental health problems (Kendler et al., 2006). Further, low self-control in the clinical cohort might have stemmed from neurobiological deficits that are prevalent in psychiatric disorders (Etkin et al., 2013). If genetic predispositions played the most dominant role in developing mental health problems and subsequent self-control problems in the clinical cohort, the associations of family functioning with self-control and mental health problems could be less salient for those adolescents. Future studies including genetic factors as moderators of the associations of family functioning with self-control and mental health problems may provide information on the context in which family functioning plays a role in the development of self-control and mental health problems.

### Associations among Self-control, Mental Health Problems, and Family Functioning from the CLPMs and RI-CLPMs

Standard CLPMs were conducted to check whether the results from the RI-CLPMs were replicated when within- and between-person effects were not separated. In the CLPMs, internalizing problems predicted subsequent self-control, but not the other way around. Self-control and externalizing problems were reciprocally associated, but the effect sizes of the cross-lagged paths from mental health problems to self-control were generally below the smallest effect size of interest. The same patterns were found in the RI-CLPM, and the effect sizes were not meaningful (below the smallest effect size of interest). In sum, the results from the CLPMs did not show patterns that are different from those of the RI-CLPMs, suggesting that, from mid-adolescence to young adulthood, self-control, mental health problems, and family functioning may not affect each other with long time lags (e.g., years).

### Shared Method Variance

The effect sizes of between-person and concurrent associations between self-control and mental health problems were much larger than the effect sizes of the associations of self-control (or mental health problems) with family functioning. Given that adolescents reported their self-control and mental health problems, whereas parents reported family functioning, the associations between self-control and mental health problems could have been inflated by the use of the same informant for both constructs (Li et al., 2019). To assess the likelihood of mono-informant bias, post hoc analyses were conducted with multiple informants. The associations between self-reported self-control and mental health problems (i.e., where adolescents reported both variables) appeared two to four times stronger than the associations between the same variables each reported by different informants (e.g., self-reported self-control and parent-reported mental health problems). Nevertheless, self-control was strongly associated with simultaneous mental health problems when self-control and mental health problems were not reported by the same informant. Thus, the strong relationships occurred not solely due to the mono-informant bias—adolescents reported both self-control and mental health problems.

### limitations and Strengths

Some limitations in the current study need to be considered for future studies. First, the findings from the current study are based on the adolescents, most of whom were ethnically Dutch and had two parents; thereby, the findings may not be generalized to fit all adolescents with different familial backgrounds. Next, it was not possible to control for another form of potential shared method variance—the measures of self-control and mental health problems were based on the same questionnaire. Therefore, it cannot be excluded that the use of different questionnaires or measures would further reduce the strengths of the associations between self-control and mental health problems. In addition, it was not possible to use multiple-indicator or factor score RI-CLPMs, and it is thus not guaranteed that each item can be interpreted in the same manner across the study waves. Furthermore, the relatively small sample size of the clinical cohort resulted in insufficient statistical power to detect within-person cross-lagged paths with small effect sizes. The results from the clinical cohort are therefore less reliable than the results from the population cohort (Button et al., 2013). Lastly, the current study did not include possible factors that may modify the associations among self-control, mental health problems, and family functioning. Future studies may examine, for example, whether different patterns of associations are found for males and females as the development of self-control and mental health problems tend to show different patterns across sex.

Notwithstanding these limitations, the current study has several strengths that render a unique contribution to the literature. First, the RI-CLPMs distinguished within-person effects from the between-person associations among self-control, mental health problems, and family functioning. This approach has the additional strength to control for unobserved stable (time-invariant) confounders by estimating stable between individual differences (Usami et al., 2019). Second, the longitudinal data with repeated measures over a period of 14 years allowed us to examine the effects across adolescence and young adulthood. Third, the robustness of the findings was checked by applying the same RI-CLPMs in a population-based as well as a clinically referred cohort and by using multiple informants.

## Conclusion

Adolescents experience vast changes, and the family is the primary social unit that helps adolescents to adapt to these developmental changes, while in turn needing to adjust to the changes in adolescents as well. This study showed stable between-individual relations among self-control, mental health problems, and family functioning among adolescents and young adults. Further, self-control problems in adolescents and young adults were concurrently associated with mental health problems at the within-individual level, even though no meaningful within-individual reciprocal associations were found between self-control, mental health problems, and family functioning. If changes in self-control, mental health problems, and family functioning in adolescents and young adults affect each other over short intervals of time, the found concurrent effects may reflect within-individual transactional effects among the constructs under the study. This calls for further research with a smaller time scale, e.g., weeks or days, at which the transactional process might take place. Moreover, the results from CLPMs replicated the nonsignificant reciprocal associations among the constructs under the current study, especially between family functioning and other constructs. It could be that the interplay between self-control, mental health problems, and family functioning occurs in earlier life, e.g., young childhood. Further research is warranted to confirm whether self-control, mental health problems, and family functioning affect each other during earlier developmental periods but not during or after adolescence.

## Supplementary Information

Supplementary information_19122021
