# Supplementary material for: Self-control, Mental Health Problems, and Family Functioning in Adolescence and Young Adulthood: Between-person Differences and Within-person Effects
Source: J Youth Adolesc. 2022 Jan 18;51(6):1181–95. doi: 10.1007/s10964-021-01564-3 (PMC9090846; doi:10.1007/s10964-021-01564-3)
Supplement: Supplementary file 1 — Supplementary information_19122021 [file 10964_2021_1564_MOESM1_ESM.docx]

**Self-control, Mental Health Problems, and Family Functioning in Adolescence and Young Adulthood: Between-person Differences and Within-person Effects. Journal of Youth and Adolescence. Y. Kim, J. S. Richards, & A. J. Oldehinkel.**

Table of Contents

[**1.** **A priori power analyses** 2](#_Toc75186953)

[**2.** **Attempted multiple indicator and factor score RI-CLPMs** 3](#_Toc75186954)

[**3.** **Self-control measure** 4](#_Toc75186955)

[**Table S1.** ASEBA Self-Control items in the YSR and ASR questionnaires 4](#_Toc75186956)

[**4.** **Pearson correlations** 5](#_Toc75186957)

[**Table S2.** Correlations between self-control, mental health problems, and family functioning in the clinical cohort 5](#_Toc75186958)

[**5.** **Population cohort RI-CLPMs** 6](#_Toc75186959)

[**Table S3.** Model fit comparisons of RI-CLPMs for self-control, internalizing problems, and family functioning in the population cohort 7](#_Toc75186960)

[**Table S4.** Model fit comparisons of RI-CLPMs for self-control, externalizing problems, and family functioning in the population cohort 8](#_Toc75186961)

[**Table S5.** Standardized path coefficients of RI-CLPMs from population cohort: self-control, mental health problems, and family functioning 9](#_Toc75186962)

[**6.** **Sensitivity analysis 1: Clinical cohort RI-CLPMs** 11](#_Toc75186963)

[**Table S6.** Standardized path coefficients of RI-CLPMs from the clinical cohort: self-control, mental health problems, and family functioning 12](#_Toc75186964)

[**7.** **Sensitivity analysis 2: Population cohort CLPMs** 14](#_Toc75186965)

[**Table S7.** Model fit comparisons of RI-CLPMs and CLPMs for self-control, mental health problems, and family functioning in the population cohort 15](#_Toc75186966)

[**Table S8.** Standardized path coefficients of CLPMs from the population cohort: self-control, mental health problems, and family functioning 16](#_Toc75186967)

[**8.** **Post hoc analyses: multiple informants RI-CLPMs** 18](#_Toc75186968)

[**Table S9.** Standardized path coefficients of RI-CLPMs with different informants: self-control, internalizing health problems, and family functioning 20](#_Toc75186969)

[**Table S10.** Standardized path coefficients of RI-CLPMs with different informants: self-control, externalizing health problems, and family functioning 22](#_Toc75186970)

1. **A priori power analyses**

**Procedure**

We conducted Monte Carlo simulations for population and clinical cohort RI-CLPMs. The expected effect sizes of the between-person associations, within-person stability, and concurrent associations are based on the results from previous research that is discussed in the present paper. No study to date has examined the associations between self-control, mental health problems, and family functioning. Thus, we set the cross-lagged effects to .10, the smallest effect size relevant for interpretation. Based on the response rates of the TRAILS, missing data patterns were considered in the power analyses: missing rates were ranging from 0% to 27.4% in the population cohort and from 0% to 25.8% in the clinical cohort.

**Results**

The sample size of 2,228 population cohort was sufficient for RI-CLPMs, with the power of .88 or higher, when the estimated between-person effects were .20 - .35, the within-person stability effects were between .30 - .46, the concurrent associations were .15 -.35, and the cross-lagged effects were .10. When the estimated effect sizes of cross-lagged paths decreased to .09, the power for some paths was below .80. Thus, we interpreted results only if the estimated effect sizes were .10 or higher, and .01 was regarded as the smallest effect size of interest.

For the clinical cohort RI-CLPMs, the sample size (N=543) was sufficient for between-person paths when the estimated effects were .20 - .35. The power was higher than .88 for the within-person stability paths (estimated effects .30-.45) and most of the concurrent associations (estimated effects .15-.35). The concurrent associations between T4 self-control and T5 mental health problems and between T5 self-control and T6 mental health problems showed a power of .64 or lower. Moreover, all cross-lagged paths showed a low power (.26 to .33) for the estimated effect size of 0.1.

1. **Attempted multiple indicator and factor score RI-CLPMs**

Based on the Hamaker’s RI-CLPM modeling (Hamaker, 2018), we fit multiple indicator RI-CLPMs using partial scalar invariant measurement models for the self-control, mental health problems, and family functioning. We constrained the variances of random intercepts and covariances at 0 for the first multiple indicator RI-CLPM. The results from the first model were not reliable because some of the standardized effect sizes were larger than 0. The unreliable results might have been due to the different range of variances of the indicators: the variances of the parcel means for externalizing problems were .03 - .07 and internalizing problems .07 - .11, which were much smaller than the variances of self-control (.13 - .49) and family functioning item scores (.23 - .63). We thus tried the same analysis again, but this time we made the variances of the internalizing and externalizing problems parcel means similar to the variance of items for other study constructs. The square root of 6 was multiplied to externalizing problems parcel means, and the square root of 4 to internalizing problems parcel means. By doing so, the variances of the indicators in the RI-CLPM were comparable across the study constructs. The second model also did not provide reliable results: some standardized effects were larger than 1. Finally, we tried to conduct the RI-CLPMs with factor scores for each construct extracted from the measurement models (from the CFA’s). The factor score RI-CLPMs did not converge when the maximum of iterations was 1000. We further increased the maximum of iterations to 10,000, but convergence was not achieved.

1. **Self-control measure**

## **Table S1.** ASEBA Self-Control items in the YSR and ASR questionnaires

| No. (YSR) | Item (ASR item different from the YSR) |
| --- | --- |
| 4 | Fail to finish things that I start (Fail to finish things that I have to do) |
| 8 | Can’t concentrate, can’t pay attention for long |
| 28 ^b^ | Breaks rules at home, school, or elsewhere |
| 41 | Impulsive or acts without thinking |
| 78^a^ | Inattentive or easily distracted (N/A) |
| 86 ^b^ | Stubborn, sullen, or irritable |
| 87 ^b^ | Sudden changes in mood or feelings |
| 95 ^b^ | Temper tantrums or hot temper |

*Note.* YSR= Youth Self-Report; ASR = Adult Self-Report.

^a^ The item excluded in the current study.

^b^ The items that are overlapping with the externalizing problems scale.

1. **Pearson correlations**

## **Table S2.** Correlations between self-control, mental health problems, and family functioning in the clinical cohort

|  | **1** | **2** | **3** | **4** | **5** | **6** | **7** | **8** | **9** | **10** | **11** | **12** | **13** | **14** | **15** | **16** | **17** | **18** | **19** |
| --- | --- | --- | --- | --- | --- | --- | --- | --- | --- | --- | --- | --- | --- | --- | --- | --- | --- | --- | --- |
| 1. SC T1 | - |  |  |  |  |  |  |  |  |  |  |  |  |  |  |  |  |  |  |
| 2. SC T2 | .499^**^ | - |  |  |  |  |  |  |  |  |  |  |  |  |  |  |  |  |  |
| 3. SC T3 | .280^**^ | .438^**^ | - |  |  |  |  |  |  |  |  |  |  |  |  |  |  |  |  |
| 4. SC T4 | .210^**^ | .338^**^ | .492^**^ | - |  |  |  |  |  |  |  |  |  |  |  |  |  |  |  |
| 5. SC T5 | .214^**^ | .309^**^ | .428^**^ | .566^**^ | - |  |  |  |  |  |  |  |  |  |  |  |  |  |  |
| 6. FF T1 | -.056 | -.065 | -.006 | -.094 | -.058 | - |  |  |  |  |  |  |  |  |  |  |  |  |  |
| 7. FF T2 | -.089 | -.005 | -.042 | -.007 | -.077 | .589^**^ | - |  |  |  |  |  |  |  |  |  |  |  |  |
| 8. FF T3 | .061 | .003 | .036 | .003 | -.072 | .492^**^ | .533^**^ | - |  |  |  |  |  |  |  |  |  |  |  |
| 9. FF T4 | -.054 | -.024 | .053 | .090 | -.030 | .421^**^ | .486^**^ | .557^**^ | - |  |  |  |  |  |  |  |  |  |  |
| 10. FF T5 | .051 | .026 | .007 | .037 | -.008 | .446^**^ | .467^**^ | .562^**^ | .588^**^ | - |  |  |  |  |  |  |  |  |  |
| 11. INT T1 | .521^**^ | .250^**^ | .066 | .058 | .161^**^ | .015 | -.022 | .075 | .049 | .092 | - |  |  |  |  |  |  |  |  |
| 12. INT T2 | .245^**^ | .461^**^ | .215^**^ | .212^**^ | .212^**^ | .010 | .054 | .110^*^ | .032 | .123^*^ | .445^**^ | - |  |  |  |  |  |  |  |
| 13. INT T3 | .165^**^ | .204^**^ | .412^**^ | .296^**^ | .282^**^ | -.015 | -.059 | .016 | .003 | .043 | .347^**^ | .484^**^ | - |  |  |  |  |  |  |
| 14. INT T4 | .059 | .145^**^ | .236^**^ | .532^**^ | .306^**^ | -.011 | .049 | .085 | .131^*^ | .118^*^ | .204^**^ | .398^**^ | .552^**^ | - |  |  |  |  |  |
| 15. INT T5 | .093 | .051 | .150^*^ | .302^**^ | .579^**^ | .069 | .076 | .099 | .056 | .175^**^ | .281^**^ | .348^**^ | .523^**^ | .568^**^ | - |  |  |  |  |
| 16. EXT T1 | .635^**^ | .321^**^ | .248^**^ | .157^**^ | .153^**^ | .010 | .014 | .115^*^ | .025 | .036 | .456^**^ | .142^**^ | .056 | -.035 | .058 | - |  |  |  |
| 17. EXT T2 | .364^**^ | .607^**^ | .341^**^ | .278^**^ | .226^**^ | .018 | .037 | .102 | .065 | .072 | .118^*^ | .256^**^ | .049 | .021 | -.032 | .448^**^ | - |  |  |
| 18. EXT T3 | .299^**^ | .387^**^ | .642^**^ | .312^**^ | .253^**^ | .059 | .052 | .112^*^ | .131^*^ | .105 | .066 | .097 | .171^**^ | .108^*^ | .03 | .323^**^ | .470^**^ | - |  |
| 19. EXT T4 | .165^**^ | .212^**^ | .358^**^ | .653^**^ | .346^**^ | -.001 | .106 | .084 | .147^**^ | .117^*^ | .040 | .101 | .172^**^ | .414^**^ | .140^*^ | .153^**^ | .311^**^ | .454^**^ | - |
| 20. EXT T5 | .150^**^ | .269^**^ | .364^**^ | .436^**^ | .589^**^ | .043 | .044 | .072 | .017 | .124^*^ | .031 | .103 | .077 | .109 | .327^**^ | .219^**^ | .286^**^ | .360^**^ | .519^**^ |

*Note*. SC = self-reported self-control problems; FF = parent-reported family functioning; INT= self-reported internalizing problems; EXT = self-reported externalizing problems. ^*^p-value <.05; ^**^ p-value<.01

# **Population cohort RI-CLPMs**

Tables S3 and S4 depict the process to identify parsimonious RI-CLPMs. The parsimonious RI-CLPM concerning internalizing problems was fitted by constraining all paths, except for the stability paths of self-control and internalizing problems and the concurrent paths between self-control and internalizing problems (Model 13 in Table S3). In the parsimonious RI-CLPM of externalizing problems, the stability paths of self-control and externalizing problems, the concurrent associations of externalizing problems with self-control problems, and the concurrent associations of externalizing problems and parent-reported family functioning were freed to vary over time, but the other paths were constrained over time (Model 12 in Table S4). The standardized coefficients for all between- and within-person associations between self-control, mental health problems, and family functioning are shown in Table S5.

## **Table S3.** Model fit comparisons of RI-CLPMs for self-control, internalizing problems, and family functioning in the population cohort

| Model | | χ^2^ (df) | RMSEA | (90% CI) | CFI | SRMR | Comparison | Δ χ^2^ (Δdf) | *p* | Constrained* |
| --- | --- | --- | --- | --- | --- | --- | --- | --- | --- | --- |
| Model 1. | Unconstrained full model | 255.900 (84) | .030 | (.026, .035) | .985 | .036 |  |  |  |  |
| Model 2. | Stability paths constrained for SC | 279.798 (88) | .041 | (.027, .035) | .983 | .040 | vs. model 1 | 23.898 (4) | <.001 | reject |
| Model 3. | Stability paths constrained for INT | 283.404 (88) | .032 | (.027, .036) | .983 | .040 | vs. model 1 | 24.277 (4) | <.001 | reject |
| Model 4. | Stability paths constrained for FF | 263.483 (88) | .030 | (.026, .034) | .984 | .038 | vs. model 1 | 8.172 (4) | .085 | accept |
| Model 5. | Model 4 + cross-lagged paths constrained for INT->SC | 267.292 (92) | .029 | (.025, .033) | .984 | .038 | vs. model 4 | 4.312 (4) | .365 | accept |
| Model 6. | Model 5 + cross-lagged paths constrained for SC->INT | 272.720 (96) | .029 | (.025, .033) | .984 | .039 | vs. model 5 | 5.336 (4) | .255 | accept |
| Model 7. | Model 6 + cross-lagged paths constrained for FF->SC | 277.915 (100) | .028 | (.024, .032) | .984 | .040 | vs. model 6 | 4.855 (4) | .302 | accept |
| Model 8. | Model 7 + cross-lagged paths constrained for SC->FF | 280.649 (104) | .028 | (.024, .032) | .984 | .040 | vs. model 7 | 1.964 (4) | .742 | accept |
| Model 9. | Model 8 + cross-lagged paths constrained for INT->FF | 284.079 (108) | .027 | (.023, .031) | .984 | .040 | vs. model 8 | 2.866 (4) | .570 | accept |
| Model 10. | Model 9 + cross-lagged paths constrained for FF->INT | 285.106 (112) | .026 | (.023, .030) | .985 | .040 | vs. model 9 | 0.738 (4) | .947 | accept |
| Model 11. | Model 10 + concurrent paths constrained for SC-INT | 299.861 (117) | .026 | (.023, .030) | .984 | .039 | vs. model 10 | 14.394 (5) | .013 | reject |
| Model 12. | Model 10 + concurrent paths constrained for FF-SC | 291.015 (117) | .026 | (.023, .030) | .985 | .041 | vs. model 10 | 6.046 (5) | .308 | accept |
| Model 13. | Model 12 + concurrent paths constrained for INT-FF | 290.732 (121) | .025 | (.021, .029) | .985 | .041 | vs. model 12 | 0.200 (5) | .999 | accept |

*Note*. SC = self-reported self-control; FF = parent-reported family functioning; INT = self-reported internalizing problems. Model 13 is used as the parsimonious RI-CLPM for the internalizing problems. * Only accepted paths are constrained; rejected paths are allowed to vary across the time.

## **Table S4.** Model fit comparisons of RI-CLPMs for self-control, externalizing problems, and family functioning in the population cohort

| Model | | χ^2^ (df) | RMSEA | (90% CI) | CFI | SRMR | Comparison | Δ χ^2^ (Δdf) | *p* | Constrained* |
| --- | --- | --- | --- | --- | --- | --- | --- | --- | --- | --- |
| Model 1. | Unconstrained full model | 267.152 (84) | .031 | (.027, .036) | .983 | .038 |  |  |  |  |
| Model 2. | Stability paths constrained for SC | 301.711 (88) | .033 | (.029, .037) | .980 | .042 | vs. model 1 | 37.060 (4) | <.001 | reject |
| Model 3. | Stability paths constrained for EXT | 279.767 (88) | .031 | (.027 .035) | .982 | .038 | vs. model 1 | 12.652 (4) | .013 | reject |
| Model 4. | Stability paths constrained for FF | 275.258 (88) | .031 | (.027, .035) | .983 | .040 | vs. model 1 | 8.693 (4) | .069 | accept |
| Model 5. | Model 4 + cross-lagged paths constrained for EXT->SC | 274.679 (92) | .030 | (.026, .034) | .983 | .039 | vs. model 4 | 3.122 (4) | .538 | accept |
| Model 6. | Model 5 + cross-lagged paths constrained for SC->EXT | 277.859 (96) | .029 | (.025, .033) | .983 | .040 | vs. model 5 | 3.374 (4) | .497 | accept |
| Model 7. | Model 6 + cross-lagged paths constrained for FF->SC | 282.355 (100) | .029 | (.025, .033) | .983 | .040 | vs. model 6 | 4.024 (4) | .403 | accept |
| Model 8. | Model 7 + cross-lagged paths constrained for SC->FF | 285.365 (104) | .028 | (.024, .033) | .983 | .040 | vs. model 7 | 1.958 (4) | .743 | accept |
| Model 9. | Model 8 + cross-lagged paths constrained for EXT->FF | 292.555 (108) | .028 | (.024, .032) | .983 | .041 | vs. model 8 | 7.172 (4) | .127 | accept |
| Model 10. | Model 9 + cross-lagged paths constrained for FF->EXT | 299.895 (112) | .027 | (.024, .031) | .982 | .041 | vs. model 9 | 7.142 (4) | .129 | accept |
| Model 11. | Model 10 + concurrent paths constrained for SC-EXT | 446.444 (117) | .036 | (.032, .039) | .969 | .056 | vs. model 10 | 91.292 (5) | <.001 | reject |
| Model 12. | Model 10 + concurrent paths constrained for FF-SC | 305.688 (117) | .027 | (.024, .031) | .982 | .042 | vs. model 10 | 5.854 (5) | .321 | accept |
| Model 13. | Model 12 + concurrent paths constrained for EXT-FF | 317.373 (122) | .027 | (.023, .030) | .982 | .042 | vs. model 12 | 11.664 (5) | .040 | reject |

*Note*. SC = self-reported self-control; FF = parent-reported family functioning; EXT = self-reported externalizing problems. Model 12 is used as the parsimonious model for the externalizing problems. * Only accepted paths are constrained; rejected paths are allowed to vary across the time.

## **Table S5.** Standardized path coefficients of RI-CLPMs from population cohort: self-control, mental health problems, and family functioning

|  |  |  |  | **Internalizing problems** | | |  | **Externalizing problems** | | |
| --- | --- | --- | --- | --- | --- | --- | --- | --- | --- | --- |
| Path |  |  | | β | s.e. | p-value |  | β | s.e. | p-value |
| Between-person | | FF-SC |  | **.223** | .04 | <.001 |  | **.210** | .04 | <.001 |
|  |  | SC-MH |  | **.709** | .025 | <.001 |  | **.753** | .024 | <.001 |
|  |  | MH-FF |  | **.217** | .039 | <.001 |  | **.217** | .038 | <.001 |
| Within-person | Stability | SC | T_1_-T_2_ | **.170** ^a^ | .029 | <.001 |  | **.158** ^a^ | .029 | <.001 |
|  |  |  | T_2_-T_3_ | **.261** ^a^ | .033 | <.001 |  | **.262** ^a^ | .032 | <.001 |
|  |  |  | T_3_-T_4_ | **.286** ^a^ | .034 | <.001 |  | **.275** ^a^ | .034 | <.001 |
|  |  |  | T_4_-T_5_ | **.343** ^a^ | .033 | <.001 |  | **.356** ^a^ | .033 | <.001 |
|  |  |  | T_5_-T_6_ | **.374** ^a^ | .031 | <.001 |  | **.405** ^a^ | .031 | <.001 |
|  |  | FF | T_1_-T_2_ | **.228** | .021 | <.001 |  | **.228** | .021 | <.001 |
|  |  |  | T_2_-T_3_ | **.269** | .025 | <.001 |  | **.268** | .025 | <.001 |
|  |  |  | T_3_-T_4_ | **.278** | .025 | <.001 |  | **.278** | .025 | <.001 |
|  |  |  | T_4_-T_5_ | **.253** | .026 | <.001 |  | **.253** | .026 | <.001 |
|  |  |  | T_5_-T_6_ | **.274** | .025 | <.001 |  | **.273** | .025 | <.001 |
|  |  | MH | T_1_-T_2_ | **.232** ^a^ | .033 | <.001 |  | **.253** ^a^ | .025 | <.001 |
|  |  |  | T_2_-T_3_ | **.321** ^a^ | .037 | <.001 |  | **.319** ^a^ | .030 | <.001 |
|  |  |  | T_3_-T_4_ | **.328** ^a^ | .040 | <.001 |  | **.261** ^a^ | .042 | <.001 |
|  |  |  | T_4_-T_5_ | **.376** ^a^ | .037 | <.001 |  | **.241** ^a^ | .059 | <.001 |
|  |  |  | T_5_-T_6_ | **.470** ^a^ | .036 | <.001 |  | **.185** ^a^ | .069 | .007 |
|  | Concurrent | FF-SC | T_1_ | **.067** | .014 | <.001 |  | **.071** | .014 | <.001 |
|  |  |  | T_2_ | **.060** | .013 | <.001 |  | **.063** | .013 | <.001 |
|  |  |  | T_3_ | **.063** | .013 | <.001 |  | **.066** | .014 | <.001 |
|  |  |  | T_4_ | **.065** | .014 | <.001 |  | **.069** | .014 | <.001 |
|  |  |  | T_5_ | **.068** | .015 | <.001 |  | **.071** | .015 | <.001 |
|  |  |  | T_6_ | **.070** | .015 | <.001 |  | **.074** | .015 | <.001 |
|  |  | SC-MH | T_1_ | **.522** ^a^ | .021 | <.001 |  | **.606** ^a^ | .018 | <.001 |
|  |  |  | T_2_ | **.455** ^a^ | .023 | <.001 |  | **.538** ^a^ | .019 | <.001 |
|  |  |  | T_3_ | **.409** ^a^ | .027 | <.001 |  | **.516** ^a^ | .021 | <.001 |
|  |  |  | T_4_ | **.524** ^a^ | .024 | <.001 |  | **.553** ^a^ | .024 | <.001 |
|  |  |  | T_5_ | **.542** ^a^ | .024 | <.001 |  | **.519** ^a^ | .025 | <.001 |
|  |  |  | T_6_ | **.566** ^a^ | .024 | <.001 |  | **.521** ^a^ | .029 | <.001 |
|  |  | MH-FF | T_1_ | **.063** | .015 | <.001 |  | **.091** ^a^ | .024 | <.001 |
|  |  |  | T_2_ | **.059** | .014 | <.001 |  | **.076** ^a^ | .024 | .002 |
|  |  |  | T_3_ | **.061** | .015 | <.001 |  | **.089** ^a^ | .026 | .001 |
|  |  |  | T_4_ | **.063** | .015 | <.001 |  | **.078** ^a^ | .030 | .009 |
|  |  |  | T_5_ | **.095** | .029 | 0.001 |  | .016 ^a^ | .029 | .591 |
|  |  |  | T_6_ | **.056** | .014 | <.001 |  | .067 ^a^ | .040 | .094 |
|  | Cross-lagged | FF-SC | T_1_-T_2_ | .023 | .015 | .113 |  | .024 | .015 | .094 |
|  |  |  | T_2_-T_3_ | .027 | .017 | .112 |  | .029 | .017 | .094 |
|  |  |  | T_3_-T_4_ | .026 | .017 | .114 |  | .028 | .017 | .095 |
|  |  |  | T_4_-T_5_ | .027 | .017 | .114 |  | .028 | .017 | .095 |
|  |  |  | T_5_-T_6_ | .028 | .018 | .111 |  | .029 | .017 | .092 |
|  |  | SC-FF | T_1_-T_2_ | .012 | .018 | .507 |  | .018 | .018 | .325 |
|  |  |  | T_2_-T_3_ | .012 | .018 | .506 |  | .019 | .019 | .324 |
|  |  |  | T_3_-T_4_ | .013 | .019 | .508 |  | .020 | .020 | .326 |
|  |  |  | T_4_-T_5_ | .012 | .018 | .507 |  | .019 | .019 | .325 |
|  |  |  | T_5_-T_6_ | .012 | .018 | .507 |  | .018 | .019 | .325 |
|  |  | SC-MH | T_1_-T_2_ | -.006 | .021 | .775 |  | **.064** | .016 | <.001 |
|  |  |  | T_2_-T_3_ | -.006 | .021 | .775 |  | **.062** | .016 | <.001 |
|  |  |  | T_3_-T_4_ | -.006 | .020 | .775 |  | **.080** | .021 | <.001 |
|  |  |  | T_4_-T_5_ | -.006 | .019 | .775 |  | **.103** | .028 | <.001 |
|  |  |  | T_5_-T_6_ | -.005 | .016 | .775 |  | **.114** | .030 | <.001 |
|  |  | MH-SC | T_1_-T_2_ | **.056** | .020 | .006 |  | **.084** | .023 | <.001 |
|  |  |  | T_2_-T_3_ | **.054** | .020 | .007 |  | **.082** | .023 | <.001 |
|  |  |  | T_3_-T_4_ | **.053** | .020 | .008 |  | **.085** | .024 | <.001 |
|  |  |  | T_4_-T_5_ | **.059** | .022 | .007 |  | **.070** | .020 | .001 |
|  |  |  | T_5_-T_6_ | **.061** | .023 | .008 |  | **.054** | .016 | .001 |
|  |  | MH-FF | T_1_-T_2_ | .028 | .019 | .137 |  | .022 | .019 | .249 |
|  |  |  | T_2_-T_3_ | .027 | .019 | .140 |  | .021 | .019 | .249 |
|  |  |  | T_3_-T_4_ | .029 | .020 | .141 |  | .024 | .021 | .249 |
|  |  |  | T_4_-T_5_ | .028 | .019 | .142 |  | .018 | .015 | .252 |
|  |  |  | T_5_-T_6_ | .031 | .021 | .140 |  | .014 | .012 | .252 |
|  |  | FF-MH | T_1_-T_2_ | .030 | .016 | .062 |  | .024 | .013 | .058 |
|  |  |  | T_2_-T_3_ | .035 | .019 | .062 |  | .026 | .014 | .058 |
|  |  |  | T_3_-T_4_ | .034 | .018 | .061 |  | .034 | .018 | .058 |
|  |  |  | T_4_-T_5_ | .030 | .016 | .062 |  | .041 | .022 | .060 |
|  |  |  | T_5_-T_6_ | .028 | .015 | .061 |  | .050 | .026 | .052 |

*Note*. β = standardized path coefficient; s.e.= standardized error.

Path coefficients in boldface are significant at p < .05

SC = self-reported self-control problems; FF = parent-reported family functioning; MH = self-reported mental health problems (i.e., internalizing and externalizing problems).

^a^ paths that are not constrained across time.

# **Sensitivity analysis 1: Clinical cohort RI-CLPMs**

The parsimonious internalizing RI-CLPM was fitted by constraining all paths to be equal over time, except for the internalizing problems stability paths, the concurrent paths between self-control problems and family functioning, and the cross-lagged paths from self-control to family functioning. In the parsimonious externalizing problems RI-CLPM, the externalizing problems stability paths, the concurrent associations between family functioning and self-control problems, the cross-lagged paths from self-control problems to family functioning were freed to vary across time whereas other paths were constrained. The standardized coefficients for all between- and within-person associations between self-control, mental health problems, and family functioning in the clinical cohort are shown in Table S6.

## **Table S6.** Standardized path coefficients of RI-CLPMs from the clinical cohort: self-control, mental health problems, and family functioning

|  |  |  |  | Internalizing problems | | |  | Externalizing problems | | |
| --- | --- | --- | --- | --- | --- | --- | --- | --- | --- | --- |
| Path |  |  |  | β | s.e. | *p*-value |  | β | s.e. | *p*-value |
| Between-person | | FF-SC |  | -.122 | .098 | .211 |  | -.087 | .107 | .416 |
|  |  | SC-MH |  | **.381** | .090 | <.001 |  | **.820** | .071 | <.001 |
|  |  | MH-FF |  | .077 | .079 | .335 |  | .147 | .090 | .103 |
| Within-person | Stability | SC | T_1_-T_2_ | **.301** | .051 | <.001 |  | **.366** | .053 | <.001 |
|  |  |  | T_2_-T_3_ | **.284** | .051 | <.001 |  | **.336** | .053 | <.001 |
|  |  |  | T_3_-T_4_ | **.309** | .057 | <.001 |  | **.331** | .059 | <.001 |
|  |  |  | T_4_-T_5_ | **.322** | .060 | <.001 |  | **.380** | .059 | <.001 |
|  |  | FF | T_1_-T_2_ | **.220** | .056 | <.001 |  | **.237** | .055 | <.001 |
|  |  |  | T_2_-T_3_ | **.183** | .052 | <.001 |  | **.201** | .054 | <.001 |
|  |  |  | T_3_-T_4_ | **.212** | .061 | .001 |  | **.234** | .063 | <.001 |
|  |  |  | T_4_-T_5_ | **.221** | .065 | .001 |  | **.238** | .066 | <.001 |
|  |  | MH | T_1_-T_2_ | **.149** ^a^ | .072 | .038 |  | **.280** | .056 | <.001 |
|  |  |  | T_2_-T_3_ | **.224** ^a^ | .081 | .006 |  | **.255** | .053 | <.001 |
|  |  |  | T_3_-T_4_ | **.356** ^a^ | .075 | <.001 |  | **.298** | .061 | <.001 |
|  |  |  | T_4_-T_5_ | **.358** ^a^ | .076 | <.001 |  | **.347** | .080 | <.001 |
|  | Concurrent | FF-SC | T_1_ | -.042 ^a^ | .051 | .409 |  | **.061** | .030 | .044 |
|  |  |  | T_2_ | **.122** ^a^ | .052 | .019 |  | **.073** | .036 | .044 |
|  |  |  | T_3_ | **.114** ^a^ | .049 | .020 |  | **.059** | .029 | .042 |
|  |  |  | T_4_ | **.163** ^a^ | .058 | .005 |  | **.057** | .029 | .047 |
|  |  |  | T_5_ | -.015 ^a^ | .068 | .826 |  | **.065** | .032 | .045 |
|  |  | SC-MH | T_1_ | **.525** | .029 | <.001 |  | **.596** ^a^ | .034 | <.001 |
|  |  |  | T_2_ | **.529** | .029 | <.001 |  | **.524** ^a^ | .042 | <.001 |
|  |  |  | T_3_ | **.516** | .026 | <.001 |  | **.548** ^a^ | .039 | <.001 |
|  |  |  | T_4_ | **.490** | .030 | <.001 |  | **.604** ^a^ | .044 | <.001 |
|  |  |  | T_5_ | **.599** | .032 | <.001 |  | **.466** ^a^ | .056 | <.001 |
|  |  | MH-FF | T_1_ | .055 | .032 | .089 |  | .044 | .027 | .099 |
|  |  |  | T_2_ | .054 | .032 | .091 |  | .052 | .031 | .097 |
|  |  |  | T_3_ | .047 | .028 | .092 |  | .042 | .025 | .100 |
|  |  |  | T_4_ | .043 | .026 | .096 |  | .048 | .029 | .101 |
|  |  |  | T_5_ | .049 | .029 | .094 |  | .067 | .041 | .100 |
|  | Cross-lagged | FF-SC | T_1_-T_2_ | .029 | .033 | .370 |  | -.010 | .034 | .769 |
|  |  |  | T_2_-T_3_ | .027 | .030 | .369 |  | -.010 | .032 | .769 |
|  |  |  | T_3_-T_4_ | .033 | .037 | .369 |  | -.011 | .036 | .769 |
|  |  |  | T_4_-T_5_ | .036 | .040 | .366 |  | -.012 | .040 | .770 |
|  |  | SC-FF | T_1_-T_2_ | -.095 ^a^ | .062 | .123 |  | -.021 | .051 | .679 |
|  |  |  | T_2_-T_3_ | .099 ^a^ | .068 | .143 |  | -.017 | .041 | .679 |
|  |  |  | T_3_-T_4_ | **.150** ^a^ | .071 | .033 |  | -.017 | .042 | .678 |
|  |  |  | T_4_-T_5_ | .116 ^a^ | .077 | .129 |  | -.018 | .044 | .679 |
|  |  | SC-MH | T_1_-T_2_ | **.105** | .043 | .014 |  | **.092** | .044 | .036 |
|  |  |  | T_2_-T_3_ | **.106** | .043 | .015 |  | **.084** | .041 | .043 |
|  |  |  | T_3_-T_4_ | **.096** | .039 | .015 |  | **.097** | .049 | .048 |
|  |  |  | T_4_-T_5_ | **.103** | .042 | .014 |  | **.132** | .062 | .034 |
|  |  | MH-SC | T_1_-T_2_ | .071 | .043 | .098 |  | -.006 | .051 | .900 |
|  |  |  | T_2_-T_3_ | .073 | .045 | .104 |  | -.006 | .047 | .900 |
|  |  |  | T_3_-T_4_ | .075 | .046 | .105 |  | -.006 | .047 | .900 |
|  |  |  | T_4_-T_5_ | .094 | .057 | .097 |  | -.006 | .045 | .899 |
|  |  | MH-FF | T_1_-T_2_ | -.008 | .044 | .857 |  | .092 | .049 | .059 |
|  |  |  | T_2_-T_3_ | -.007 | .041 | .857 |  | .074 | .041 | .068 |
|  |  |  | T_3_-T_4_ | -.007 | .040 | .857 |  | .077 | .043 | .070 |
|  |  |  | T_4_-T_5_ | -.009 | .047 | .857 |  | .069 | .039 | .074 |
|  |  | FF-MH | T_1_-T_2_ | -.012 | .034 | .730 |  | -.024 | .030 | .438 |
|  |  |  | T_2_-T_3_ | -.012 | .034 | .730 |  | -.022 | .029 | .433 |
|  |  |  | T_3_-T_4_ | -.012 | .034 | .730 |  | -.030 | .038 | .437 |
|  |  |  | T_4_-T_5_ | -.013 | .039 | .731 |  | -.039 | .051 | .443 |

*Note*. β = standardized path coefficient; s.e.= standardized error.

Path coefficients in boldface are significant at p < .05

SC = self-reported self-control problems; FF = parent-reported family functioning; MH = self-reported mental health problems (i.e., internalizing and externalizing problems.

^a^ paths that are not constrained across time.

1. **Sensitivity analysis 2: Population cohort CLPMs**

**Procedure**

The traditional CLPM is equivalent to the RI-CLPM with all random intercepts are fixed to zero. Thus, the CLPM is nested in the RI-CLPM, so we can compare model fit indices. The CLPMs corresponding to the population RI-CLPMs were fitted by excluding between-person latent variables. After that, the models were compared using the Satorra-Bentler scaled chi-square difference test (Satorra & Bentler, 2001). RMSEA, CFI, and SRMR were also used to assess model fit.

**Results**

The fit of the CLPMs and the RI-CLPMs self-control, mental health problems, and family functioning is depicted in Table S7. Both internalizing and externalizing CLPMs were significantly worse than the RI-CLPMs according to the Satorra-Bentler scaled chi-square difference test. Other fit indices also indicated a better fit of the RI-CLPMs compared to the CLPMs. Standardized path coefficients of the CLPMs are shown in Table S8.

## **Table S7.** Model fit comparisons of RI-CLPMs and CLPMs for self-control, mental health problems, and family functioning in the population cohort

|  | χ^2^ (df) | Δ χ^2^ (Δdf) | RMSEA | (90% CI) | ΔRMSEA | CFI | ΔCFI | SRMR | ΔSRMR |
| --- | --- | --- | --- | --- | --- | --- | --- | --- | --- |
| Internalizing problems |  |  |  |  |  |  |  |  |  |
| RI-CLPM | 290.732 (121) |  | .025 | (.021, .029) |  | .985 |  | .041 |  |
| CLPM^a^ | 959.97 (127) | 532.202* (6) | .054 | (.051, .057) | .029 | .926 | -.059 | .088 | .047 |
| Externalizing problems |  |  |  |  |  |  |  |  |  |
| RI-CLPM | 305.688 (117) |  | .027 | (.024 .031) |  | .982 |  | .042 |  |
| CLPM^a^ | 945.629 (123) | 546.405* (6) | .055 | (.052, .058) | .028 | .923 | -.059 | .088 | .046 |

*Note*. ^a^ Model comparisons were made with the respective RI-CLPM model. * *p*<.001

## **Table S8.** Standardized path coefficients of CLPMs from the population cohort: self-control, mental health problems, and family functioning

|  |  |  | Internalizing problems | | |  | Externalizing problems | | |
| --- | --- | --- | --- | --- | --- | --- | --- | --- | --- |
| Path |  | | β | s.e. | p-value |  | β | s.e. | p-value |
| Stability | SC | T_1_-T_2_ | **.365** ^a^ | .019 | <.001 |  | **.353** ^a^ | .020 | <.001 |
|  |  | T_2_-T_3_ | **.457** ^a^ | .020 | <.001 |  | **.459** ^a^ | .020 | <.001 |
|  |  | T_3_-T_4_ | **.472** ^a^ | .020 | <.001 |  | **.467** ^a^ | .020 | <.001 |
|  |  | T_4_-T_5_ | **.512** ^a^ | .020 | <.001 |  | **.527** ^a^ | .020 | <.001 |
|  |  | T_5_-T_6_ | **.536** ^a^ | .020 | <.001 |  | **.571** ^a^ | .020 | <.001 |
|  | FF | T_1_-T_2_ | **.503** | .014 | <.001 |  | **.504** | .014 | <.001 |
|  |  | T_2_-T_3_ | **.547** | .016 | <.001 |  | **.547** | .016 | <.001 |
|  |  | T_3_-T_4_ | **.569** | .016 | <.001 |  | **.571** | .016 | <.001 |
|  |  | T_4_-T_5_ | **.552** | .018 | <.001 |  | **.554** | .018 | <.001 |
|  |  | T_5_-T_6_ | **.571** | .015 | <.001 |  | **.574** | .015 | <.001 |
|  | MH | T_1_-T_2_ | **.476** ^a^ | .020 | <.001 |  | **.376** ^a^ | .020 | <.001 |
|  |  | T_2_-T_3_ | **.568** ^a^ | .019 | <.001 |  | **.456** ^a^ | .022 | <.001 |
|  |  | T_3_-T_4_ | **.573** ^a^ | .019 | <.001 |  | **.443** ^a^ | .024 | <.001 |
|  |  | T_4_-T_5_ | **.577** ^a^ | .021 | <.001 |  | **.468** ^a^ | .026 | <.001 |
|  |  | T_5_-T_6_ | **.638** ^a^ | .021 | <.001 |  | **.471** ^a^ | .031 | .007 |
| Concurrent | FF-SC | T_1_ | **.065** | .010 | <.001 |  | **.067** ^a^ | .010 | <.001 |
|  |  | T_2_ | **.072** | .011 | <.001 |  | **.074** ^a^ | .011 | <.001 |
|  |  | T_3_ | **.076** | .012 | <.001 |  | **.078** ^a^ | .012 | <.001 |
|  |  | T_4_ | **.079** | .012 | <.001 |  | **.081** ^a^ | .012 | <.001 |
|  |  | T_5_ | **.085** | .013 | <.001 |  | **.087** ^a^ | .013 | <.001 |
|  |  | T_6_ | **.088** | .014 | <.001 |  | **.091** ^a^ | .014 | <.001 |
|  | SC-MH | T_1_ | **.559** ^a^ | .015 | <.001 |  | **.630** | .014 | <.001 |
|  |  | T_2_ | **.492** ^a^ | .018 | <.001 |  | **.552** | .016 | <.001 |
|  |  | T_3_ | **.440** ^a^ | .021 | <.001 |  | **.524** | .019 | <.001 |
|  |  | T_4_ | **.537** ^a^ | .021 | <.001 |  | **.565** | .021 | <.001 |
|  |  | T_5_ | **.559** ^a^ | .022 | <.001 |  | **.523** | .022 | <.001 |
|  |  | T_6_ | **.564** ^a^ | .023 | <.001 |  | **.528** | .024 | <.001 |
|  | MH-FF | T_1_ | **.062** | .010 | <.001 |  | **.101** | .018 | <.001 |
|  |  | T_2_ | **.073** | .012 | <.001 |  | **.077** | .021 | <.001 |
|  |  | T_3_ | **.077** | .013 | <.001 |  | **.091** | .023 | <.001 |
|  |  | T_4_ | **.081** | .014 | <.001 |  | **.078** | .026 | .002 |
|  |  | T_5_ | **.105** | .026 | <.001 |  | .037 | .026 | .161 |
|  |  | T_6_ | **.076** | .013 | <.001 |  | **.073** | .035 | .04 |
| Cross-lagged | FF-SC | T_1_-T_2_ | **.039** | .009 | <.001 |  | **.040** | .009 | <.001 |
|  |  | T_2_-T_3_ | **.042** | .010 | <.001 |  | **.044** | .010 | <.001 |
|  |  | T_3_-T_4_ | **.043** | .010 | <.001 |  | **.045** | .010 | <.001 |
|  |  | T_4_-T_5_ | **.045** | .011 | <.001 |  | **.047** | .011 | <.001 |
|  |  | T_5_-T_6_ | **.044** | .011 | <.001 |  | **.046** | .011 | <.001 |
|  | SC-FF | T_1_-T_2_ | .019 | .012 | .104 |  | **.025** | .012 | .045 |
|  |  | T_2_-T_3_ | .019 | .012 | .103 |  | **.025** | .013 | .044 |
|  |  | T_3_-T_4_ | .020 | .012 | .104 |  | **.026** | .013 | .044 |
|  |  | T_4_-T_5_ | .020 | .012 | .104 |  | **.026** | .013 | .044 |
|  |  | T_5_-T_6_ | .019 | .012 | .105 |  | **.025** | .013 | .044 |
|  | SC-MH | T_1_-T_2_ | **.026** | .012 | .027 |  | **.093** | .011 | <.001 |
|  |  | T_2_-T_3_ | **.026** | .012 | .028 |  | **.090** | .010 | <.001 |
|  |  | T_3_-T_4_ | **.027** | .012 | .028 |  | **.112** | .013 | <.001 |
|  |  | T_4_-T_5_ | **.026** | .012 | .028 |  | **.137** | .016 | <.001 |
|  |  | T_5_-T_6_ | **.022** | .010 | .028 |  | **.143** | .017 | <.001 |
|  | MH-SC | T_1_-T_2_ | **.106** | .012 | <.001 |  | **.098** | .015 | <.001 |
|  |  | T_2_-T_3_ | **.105** | .012 | <.001 |  | **.096** | .015 | <.001 |
|  |  | T_3_-T_4_ | **.108** | .013 | <.001 |  | **.103** | .016 | <.001 |
|  |  | T_4_-T_5_ | **.113** | .013 | <.001 |  | **.089** | .014 | <.001 |
|  |  | T_5_-T_6_ | **.116** | .014 | <.001 |  | **.072** | .011 | <.001 |
|  | MH-FF | T_1_-T_2_ | **.038** | .011 | .001 |  | .026 | .014 | .051 |
|  |  | T_2_-T_3_ | **.038** | .011 | .001 |  | .026 | .013 | .051 |
|  |  | T_3_-T_4_ | **.040** | .012 | .001 |  | .028 | .014 | .051 |
|  |  | T_4_-T_5_ | **.039** | .012 | .001 |  | .023 | .012 | .053 |
|  |  | T_5_-T_6_ | **.042** | .012 | .001 |  | .019 | .010 | .053 |
|  | FF-MH | T_1_-T_2_ | **.042** | .010 | <.001 |  | **.035** | .008 | <.001 |
|  |  | T_2_-T_3_ | **.045** | .010 | <.001 |  | **.036** | .009 | <.001 |
|  |  | T_3_-T_4_ | **.047** | .011 | <.001 |  | **.045** | .011 | <.001 |
|  |  | T_4_-T_5_ | **.044** | .010 | <.001 |  | **.054** | .013 | <.001 |
|  |  | T_5_-T_6_ | **.040** | .009 | <.001 |  | **.060** | .014 | <.001 |

*Note*. β = standardized path coefficient; s.e.= standardized error.

Path coefficients in boldface are significant at p < .05

SC = self-reported self-control problems; FF = parent-reported family functioning; MH = self-reported mental health problems (i.e., internalizing and externalizing problems).

^a^ paths that are not constrained across time.

1. **Post hoc analyses: multiple informants RI-CLPMs**

**Measures**

In addition to the parent-reported family functioning, and self-reported self-control and mental health problems, parent-reported self-control and mental health problems and teacher-reported mental health problems were included in the post hoc RI-CLPMs.

***Parent-reported self-control and mental health problems (T_1_ to T_3_).*** Parent-reported self-control and internalizing and externalizing problems were assessed by the Child Behaviour Checklist items (CBCL). The content of each item in the CBCL for self-control and mental health problems was comparable to the items from the Youth Self-Report (YSR) which was used to assess self-reported self-control and mental health problems. We used the items that correspond to the self-reported self-control, internalizing, and externalizing problem measures that are used in the population cohort RI-CLPMs. Participants rated the items from 0 (not true) to 2 (very or often true). A higher score indicates more problems.

***Teacher-reported mental health problems (T_1_ to T_3_).*** The Teacher’s Checklist of Psychopathology (TCP) was used to assess teacher-reported mental health problems. The TCP includes composite items describing dimensions of CBCL/YSR. The dimensions of withdrawn/depressed, anxious/depressed, and physical complaints correspond to internalizing problems, and aggressive and rule-breaking behaviors correspond to externalizing problems. Participants could rate from 0 (not applicable) to 4 (very clearly or frequently applicable). The rating was recoded to be consistent with the coding of the YSR and CBCL. Mean scores of the dimensions were used to indicate internalizing and externalizing problems.

**Procedures**

Post hoc RI-CLPMs were fitted for the study constructs across T_1_ to T_3_ since parent- or teacher-reported self-control and mental health problems were only assessed from T_1_ to T_3_. Because family functioning was only reported by parents, in a total of six post hoc analyses was conducted based on the following combinations of informants for self-control and mental health problems: self-reported self-control and internalizing problems, self-reported self-control and parent-reported mental health problems, self-reported self-control and teacher-reported mental health problems, parent-reported self-control and self-reported mental health problems, parent-reported self-control and mental health problems, and parent-reported self-control and teacher-reported mental health problems. For each model, a parsimonious RI-CLPM was fitted following the procedure described in section 5.

**Results**

Tables S9 and S10 show the standardized path coefficients for each model with different informants for self-control and mental health problems.

## **Table S9.** Standardized path coefficients of RI-CLPMs with different informants: self-control, internalizing health problems, and family functioning

|  | | | | 1.SR-SC & SR-INT | 2.SR-SC & PR-INT | 3.SR-SC & TR-INT | 4.PR-SC & SR-INT | 5.PR-SC & PR-INT | 6.PR-SC & TR-INT |
| --- | --- | --- | --- | --- | --- | --- | --- | --- | --- |
| Path | | |  | β (s.e.) | β (s.e.) | β (s.e.) | β (s.e.) | β (s.e.) | β (s.e.) |
| Between-person |  | FF-SC |  | **.144** (.06) | **.153** (.06) | **.123** (.07) | **.361** (.04) | **.361** (.04) | **.363** (.04) |
|  |  | SC-INT |  | **.626** (.05) | **.238** (.06) | -.121 (.16) | **.129** (.06) | **.558** (.03) | **.294** (.07) |
|  |  | INT-FF |  | **.134** (.07) | **.363** (.05) | -.018 (.1) | .121 (.07) | **.371** (.04) | .039 (.09) |
| Within-person | | |  |  |  |  |  |  |  |
|  | Stability | SC | T_1_-T_2_ | .043 ^a^ (.06) | **.157** ^a^ (.05) | **.182** ^a^ (.05) | **.139** (.07) | .128 (.07) | **.148** (.07) |
|  |  |  | T_2_-T_3_ | **.275** ^a^ (.04) | **.304** ^a^ (.04) | **.324** ^a^ (.04) | .120 (.07) | .115 (.06) | .129 (.07) |
|  |  | FF | T_1_-T_2_ | **.193** (.04) | **.178** (.04) | **.195** (.04) | **.178** (.04) | **.174** (.04) | **.181** (.04) |
|  |  |  | T_2_-T_3_ | **.238** (.05) | **.219** (.05) | **.239** (.05) | **.215** (.05) | **.210** (.05) | **.218** (.05) |
|  |  | INT | T_1_-T_2_ | **.321** (.04) | **.151** (.07) | **.118** (.05) | **.253** ^a^ (.05) | **.150** (.06) | **.106** (.05) |
|  |  |  | T_2_-T_3_ | **.326** (.05) | .136 (.07) | **.138** (.06) | **.384** ^a^ (.04) | **.140** (.07) | **.125** (.06) |
|  | Concurrent | FF-SC | T_1_ | **.102** (.03) | **.099** (.03) | **.112** (.03) | **.201** (.03) | **.206** (.03) | **.202** (.03) |
|  |  |  | T_2_ | **.082** (.02) | **.081** (.02) | **.093** (.03) | **.201** (.03) | **.198** (.03) | **.201** (.03) |
|  |  |  | T_3_ | **.082** (.02) | **.083** (.03) | **.096** (.03) | **.201** (.03) | **.199** (.03) | **.201** (.03) |
|  |  | SC-INT | T_1_ | **.506** (.02) | **.120** ^a^ (.04) | **.154** (.04) | **.122** (.03) | **.404** (.03) | **.088** (.04) |
|  |  |  | T_2_ | **.482** (.02) | **.238** ^a^ (.05) | **.135** (.03) | **.145** (.03) | **.485** (.03) | **.090** (.04) |
|  |  |  | T_3_ | **.473** (.02) | **.160** ^a^ (.04) | **.142** (.04) | **.144** (.03) | **.481** (.03) | **.092** (.04) |
|  |  | INT-FF | T_1_ | **.103** (.03) | **.115** ^a^ (.05) | **.149** (.04) | **.105** (.03) | **.118** ^a^ (.04) | **.130** (.04) |
|  |  |  | T_2_ | **.087** (.03) | **.241** ^a^ (.05) | **.114** (.03) | **.092** (.03) | **.223** ^a^ (.04) | **.099** (.03) |
|  |  |  | T_3_ | **.090** (.03) | **.223** ^a^ (.04) | **.121** (.03) | **.095** (.03) | **.218** ^a^ (.03) | **.103** (.03) |
|  | Cross-lagged | FF-SC | T_1_-T_2_ | .027 (.03) | .015 (.03) | .035 (.03) | **.089** (.04) | **.085** (.03) | **.092** (.04) |
|  |  |  | T_2_-T_3_ | .031 (.03) | .018 (.03) | .042 (.03) | **.104** (.04) | **.101** (.04) | **.108** (.04) |
|  |  | SC-FF | T_1_-T_2_ | .003 (.03) | .026 (.03) | .037 (.03) | .077 (.04) | .068 (.04) | .077 (.04) |
|  |  |  | T_2_-T_3_ | .003 (.04) | .027 (.03) | .04 (.03) | .068 (.04) | .062 (.04) | .069 (.04) |
|  |  | SC-INT | T_1_-T_2_ | **-.090** ^a^ (.04) | .071 ^a^ (.04) | .067 (.04) | .024 (.04) | .030 (.05) | -.023 (.05) |
|  |  |  | T_2_-T_3_ | **.077** ^a^ (.04) | **.174** ^a^ (.05) | .073 (.05) | .021 (.03) | .026 (.04) | -.021 (.04) |
|  |  | INT-SC | T_1_-T_2_ | **.160** ^a^ (.05) | **.095** (.04) | **.072** (.04) | .080 (.04) | .076 (.05) | .017 (.04) |
|  |  |  | T_2_-T_3_ | .058 ^a^ (.04) | **.085** (.03) | **.079** (.04) | .080 (.04) | .071 (.04) | .020 (.04) |
|  |  | INT-FF | T_1_-T_2_ | .068 (.04) | **.078** (.04) | **.072** (.03) | **.069** (.03) | .043 (.04) | .053 (.03) |
|  |  |  | T_2_-T_3_ | .072 (.04) | **.073** (.04) | **.083** (.04) | **.071** (.04) | .041 (.04) | .061 (.04) |
|  |  | FF-INT | T_1_-T_2_ | .043 (.03) | .007 ^a^ (.05) | **.087** (.04) | .046 (.03) | .011 ^a^ (.04) | .079 (.04) |
|  |  |  | T_2_-T_3_ | .050 (.03) | **.150** ^a^ (.05) | **.110** (.04) | .053 (.03) | **.140** ^a^ (.04) | **.097** (.04) |

*Note*. β = standardized path coefficient; s.e.= standardized error.

Path coefficients in boldface are significant at p < .05

SR = self-reported; PR = parent-reported; TR = teacher-reported; SC = self-control problems; FF = (parent-reported) family functioning; INT = internalizing problems

^a^ paths that are not constrained across time.

## **Table S10.** Standardized path coefficients of RI-CLPMs with different informants: self-control, externalizing health problems, and family functioning

|  | | | | 1.SR-SC & SR-EXT | 2.SR-SC & PR-EXT | 3.SR-SC & TR-EXT | 4.PR-SC & SR-EXT | 5.PR-SC & PR-EXT | 6.PR-SC & TR-EXT |
| --- | --- | --- | --- | --- | --- | --- | --- | --- | --- |
| Path | | |  | β (s.e.) | β (s.e.) | β (s.e.) | β (s.e.) | β (s.e.) | β (s.e.) |
| Between-person |  | FF-SC |  | **.144** (.06) | .124 (.07) | **.143** (.07) | **.357** (.05) | **.379** (.04) | **.363** (.05) |
|  |  | SC-EXT |  | **.674** (.05) | **.295** (.07) | **.222** (.09) | **.376** (.07) | **.925** (.02) | **.529** (.06) |
|  |  | EXT-FF |  | **.193** (.07) | **.356** (.05) | **.168** (.07) | **.188** (.08) | **.376** (.04) | **.179** (.08) |
| Within-person | | |  |  |  |  |  |  |  |
|  | Stability | SC | T_1_-T_2_ | **.099** ^a^ (.05) | **.178** ^a^ (.05) | **.171** ^a^ (.05) | **.166** (.07) | **.197** (.06) | **.166** (.06) |
|  |  |  | T_2_-T_3_ | **.260** ^a^ (.04) | **.323** ^a^ (.04) | **.319** ^a^ (.04) | **.151** (.07) | **.167** (.06) | **.146** (.06) |
|  |  | FF | T_1_-T_2_ | **.193** (.04) | **.171** (.04) | **.193** (.04) | **.180** (.04) | **.170** (.04) | **.180** (.04) |
|  |  |  | T_2_-T_3_ | **.237** (.05) | **.208** (.05) | **.237** (.05) | **.220** (.05) | **.205** (.05) | **.216** (.05) |
|  |  | EXT | T_1_-T_2_ | **.150** ^a^ (.05) | **.224** ^a^ (.08) | .108 (.07) | **.216** ^a^ (.05) | **.259** ^a^ (.09) | **.143** (.06) |
|  |  |  | T_2_-T_3_ | **.284** ^a^ (.04) | .010 ^a^ (.12) | .140 (.09) | **.320** ^a^ (.05) | .059 ^a^ (.12) | **.193** (.08) |
|  | Concurrent | FF-SC | T_1_ | **.107** (.03) | **.111** (.03) | **.109** (.03) | **.206** (.03) | **.190** (.03) | **.204** (.03) |
|  |  |  | T_2_ | **.085** (.02) | **.092** (.02) | **.089** (.03) | **.205** (.03) | **.196** (.03) | **.199** (.03) |
|  |  |  | T_3_ | **.088** (.02) | **.095** (.03) | **.092** (.03) | **.211** (.03) | **.201** (.03) | **.206** (.03) |
|  |  | SC-EXT | T_1_ | **.602** (.02) | **.177** (.03) | **.139** (.04) | **.246** (.03) | **.592** ^a^ (.03) | **.165** (.04) |
|  |  |  | T_2_ | **.571** (.02) | **.239** (.04) | **.116** (.03) | **.302** (.03) | **.458** ^a^ (.06) | **.160** (.04) |
|  |  |  | T_3_ | **.545** (.02) | **.209** (.04) | **.129** (.04) | **.288** (.03) | **.544** ^a^ (.04) | **.185** (.04) |
|  |  | EXT-FF | T_1_ | **.104** (.03) | **.243** (.03) | .071 (.04) | **.104** (.03) | **.236** (.03) | .064 (.04) |
|  |  |  | T_2_ | **.088** (.03) | **.287** (.04) | .051 (.03) | **.094** (.03) | **.278** (.04) | .046 (.03) |
|  |  |  | T_3_ | **.085** (.03) | **.248** (.03) | .058 (.04) | **.090** (.03) | **.231** (.03) | .053 (.04) |
|  | Cross-lagged | FF-SC | T_1_-T_2_ | .029 (.03) | .024 (.03) | .033 (.03) | **.083** (.03) | .058 ^a^ (.04) | **.085** (.04) |
|  |  |  | T_2_-T_3_ | .035 (.03) | .029 (.03) | .039 (.03) | **.101** (.04) | **.151** ^a^ (.05) | **.100** (.04) |
|  |  | SC-FF | T_1_-T_2_ | .040 (.04) | .031 (.03) | .040 (.03) | **.093** (.04) | .040 (.04) | **.087** (.04) |
|  |  |  | T_2_-T_3_ | .044 (.04) | .033 (.03) | .042 (.03) | **.085** (.04) | .034 (.03) | **.078** (.04) |
|  |  | SC-EXT | T_1_-T_2_ | **.091** (.04) | **.107** ^a^ (.04) | .054 (.04) | **.104** (.04) | **-.124** (.06) | .078 (.05) |
|  |  |  | T_2_-T_3_ | **.089** (.03) | **.201** ^a^ (.06) | .062 (.05) | **.085** (.04) | **-.088** (.04) | .078 (.05) |
|  |  | EXT-SC | T_1_-T_2_ | **.110** (.04) | .080 (.05) | .013 (.04) | **.151** (.04) | -.023 ^a^ (.07) | .012 ^a^ (.05) |
|  |  |  | T_2_-T_3_ | **.109** (.04) | .060 (.04) | .015 (.04) | **.151** (.04) | -.185 ^a^ (.11) | **.232** ^a^ (.06) |
|  |  | EXT-FF | T_1_-T_2_ | -.004 (.04) | **.125** (.05) | .011 (.04) | .051 ^a^ (.04) | .082 (.05) | .005 (.04) |
|  |  |  | T_2_-T_3_ | -.004 (.04) | **.096** (.04) | .013 (.04) | -.056 ^a^ (.04) | .063 (.04) | .005 (.04) |
|  |  | FF-EXT | T_1_-T_2_ | .034 (.03) | **.147** (.04) | .034 (.04) | .024 (.03) | **.153 (.04)** | .020 (.04) |
|  |  |  | T_2_-T_3_ | .037 (.03) | **.155** (.04) | .046 (.05) | .026 (.03) | **.155 (.04)** | .027 (.05) |

*Note*. β = standardized path coefficient; s.e.= standardized error.

Path coefficients in boldface are significant at p < .05

SR = self-reported; PR = parent-reported; TR = teacher-reported; SC = self-control problems; FF = (parent-reported) family functioning; EXT = externalizing problems

^a^ paths that are not constrained across time.

**References**

Hamaker, E. (November 2018). How to run a multiple indicator RI-CLPM with Mplus. Retrieved from <http://www.statmodel.com/download/RI-CLPM.pdf>

Kline, R. B. (2015). *Principles and practice of structural equation modeling.* Guilford publications.

Satorra, A., & Bentler, P.M. (2001). A scaled difference chi-square test statistic for moment structure analysis. *Psychometrika, 66*(4), 507-514.
